# Molecular Aspects of Tannin-Anthelmintic Interactions as Revealed by NMR Spectroscopy

**DOI:** 10.1021/acsomega.5c03937

**Published:** 2025-07-15

**Authors:** Mimosa Sillanpää, Petri Tähtinen, Maarit Karonen

**Affiliations:** Department of Chemistry, 8058 University of Turku, FI-20014 Turku, Finland

## Abstract

The interactions between plant polyphenols and commercial anthelmintics remain largely unexplored, despite the benefits of understanding these interactions for mitigating anthelmintic resistance. This study investigated the interactions of flavan-3-ols, dimeric proanthocyanidins, and selected hydrolyzable tannins with two anthelmintics, ivermectin (IVM) and thiabendazole (TBZ), at various polyphenol to anthelmintic molar ratios using nuclear magnetic resonance (NMR) spectroscopy. Chemical shift changes (Δ*δ*s), indicating interaction, were observed for specific regions of the compounds and at all polyphenol to anthelmintic molar ratios. For IVM interactions with polyphenols, downfield Δ*δ*s were observed and were primarily associated with hydroxyl groups within the structures of the anthelmintic and polyphenols, suggesting that the interactions involved hydrogen bond formation. For TBZ interactions with polyphenols, however, both upfield and downfield Δ*δ*s were observed, suggesting that both hydrogen bonding and hydrophobic interactions were involved. All of the studied polyphenols interacted more strongly with TBZ than with IVM.

## Graphical abstract

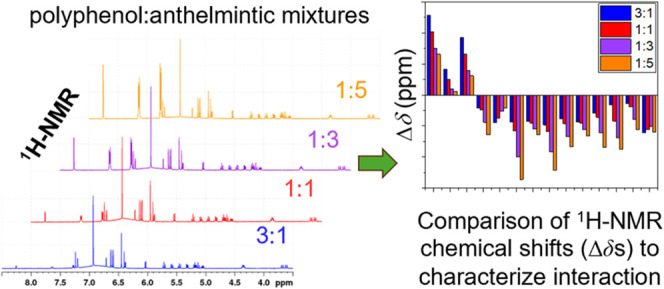

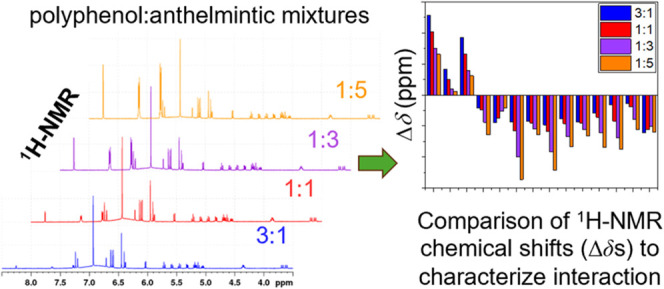

## 1 Introduction

There is a rising demand for novel strategies to counteract gastrointestinal nematodes because of the ever-increasing threat of anthelmintic resistance to animal health and livestock production.^1–5^ One proposed strategy is to use a combination of two or more nematode control methods, e.g., rotational grazing with coadministration of multiple anthelmintic compounds,^6,7^ including commercial and natural anthelmintics such as plant polyphenols.^8–10^ Plant polyphenols include a wide range of different compounds with antioxidant, antimicrobial, and anthelmintic properties.^11–18^ They can be classified into flavonoids, stilbenoids, lignans, and tannins, with the last group comprising subgroups of phlorotannins, proanthocyanidins (PAs, syn. condensed tannins), and hydrolyzable tannins (HTs). Of these, many PAs and HTs have been identified as compounds of interest because of their beneficial effects on animal health when added to animal feed, such as lower parasitic infection pressure, lower emission of greenhouse gases during rumination, and higher uptake of dietary proteins resulting in increased milk production.^8,9,17–22^

PAs and HTs are structurally different and are produced by different biosynthetic pathways. PAs are oligomers and polymers formed of flavan-3-ol monomeric units, most commonly of (epi)­catechin and (epi)­gallocatechin units. These units are linked together via C-4–C-8 or C-4–C-6 carbon–carbon bonds (Figure 1). PAs incorporating only the aforementioned C–C linkages are referred to as B-type PAs. These highly diverse compounds can also contain an additional interflavanoid ether bond between C-2 and either C-5 or C-7, and PAs with these types of linkages are referred to as A-type PAs. Monomeric HTs, on the other hand, are composed of a central polyol esterified to different variants of galloyl groups. HTs can be further divided into simple gallic acid derivatives, gallotannins (GTs) and ellagitannins (ETs). Simple gallic acid derivates consist of galloyl groups esterified to a central polyol, most commonly glucose, while gallotannins consist of additional galloyl groups linked via meta-depside bonds to the galloyls directly attached to glucose. In ETs, two or more of the gallic acid moieties attached to the polyol are linked together by intramolecular C–C bonds, forming hexahydroxydiphenoyl (HHDP) or nonahydroxytriphenoyl (NHTP) groups or their further oxidized forms. The central polyol core can be cyclic or acyclic and oligomerization can occur through different types of intermolecular linkages between monomeric ET units, contributing to the structural variety of ETs.

**Figure 1:**
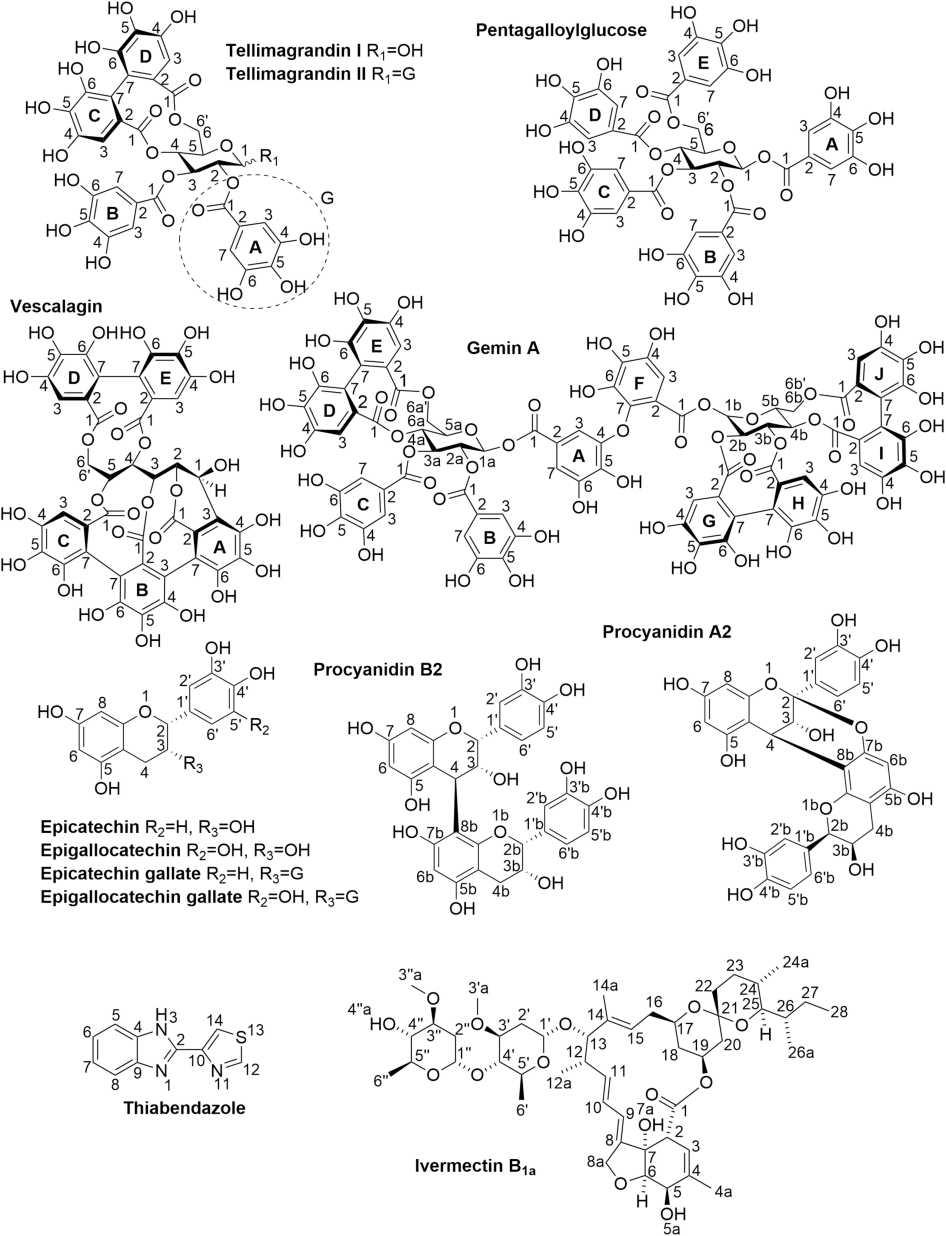
Polyphenols and commercial anthelmintics used in the study.

Certain structural features of PAs and HTs, e.g., degree of polymerization and varying number of galloyl groups, have been linked to their anthelmintic properties. Their levels in tannin-rich forages are positively linked to the beneficial effects of these forages, with the effects being highly dose dependent.^21,23–25^ The effect of administering combinations of commercial and natural anthelmintics on anthelmintic-resistant nematodes has been tested in vitro and in vivo, but the results have been contradictory. Positive effects were reported when ivermectin (IVM) was administered with tannin-rich forage from redberry juniper (*Juniperus pinchotii*) both in vivo and in vitro.^26,27^ Conversely, the administration of IVM with sainfoin (*Onobrychis viciifolia*) had the opposite effect, probably because PAs in sainfoin pellets complexed with IVM in vitro, leading to lower pharmacokinetics seen in vivo.^28,29^ On the other hand, in other in vitro studies, PAs and monoterpenes increased the efficacy of other types of commercial anthelmintics.^30–32^

These studies highlight the necessity for a deeper understanding of how different types of anthelmintic compounds interact with polyphenols. As the beneficial effects of these compounds appear to vary depending on the tannin composition and the commercial anthelmintic employed, the interactions of PAs and HTs with model substances from two widely used broad-spectrum anthelmintic classes, macrocyclic lactones (IVM) and benzimidazoles (thiabendazole, TBZ), were studied. Although these two classes of tannins and TBZ have previously been shown to interact using isothermal titration calorimetry (ITC), their interactions have not been studied using nuclear magnetic resonance (NMR) spectroscopy, which unlike ITC, can deliver detailed information on intermolecular interactions by measuring chemical shift perturbations (CSPs) arising from changes in the local environments of the interacting nuclei.^33–35^ These changes can be observed as chemical shift changes (Δ*δ*s) in the NMR spectrum, with the magnitudes and directions of the changes yielding information on the strengths and mechanisms of the interaction.^35^ This kind of approach has previously been used to study different interactions of tannins with, e.g., proteins, lipids, and crude drug constituents, revealing the sites of interactions and providing information on the reaction stoichiometries and affinity constants.^11,36–41^

In this study, the interactions of various flavan-3-ols, dimeric PAs, and HTs with two commercial anthelmintics, IVM and TBZ, were studied by NMR spectroscopy across different polyphenol to anthelmintic molar ratios (Figure 1). The observed chemical shifts (*δ*) in the ^1^H NMR spectra of the tannin-anthelmintic mixtures were compared with those of the corresponding signals of the pure compounds, with the observed effects in the *δs* of the mixtures ranging from no changes to clear shift changes across the molar ratio range.

## 2 Results and Discussion

This study used NMR spectroscopy to examine interactions between selected flavan-3-ols, dimeric PAs, and HTs with the anthelmintics IVM and TBZ across varying molar ratios. The ^1^H NMR signals of each compound were assigned as described in the Materials and Methods section and the assignments for each compound are presented in Figures S1–S13. The results indicated distinct interaction mechanisms between the polyphenols and the anthelmintics. For most interacting species, changes in the polyphenol to anthelmintic molar ratio produced systematic Δ*δ*s, indicating a direct interaction or some conformational or environmental change associated with the interaction.^37^ As these Δ*δ*s were not observed for all signals of the polyphenols, the changes cannot stem simply from the effect produced when one compound was added to the other, but must be due to interactions between the polyphenols and anthelmintics. The individual proton signals of the polyphenol-anthelmintic mixtures were classified into three categories: (1) signals with no Δ*δ* observed when compared with the chemical shifts of the pure compound; (2) signals with Δ*δ*s observed when compared with the chemical shifts of the pure compound, but no systematic Δ*δ*s within the molar ratio range; and (3) signals with systematic Δ*δ*s observed within the molar ratio range as the anthelmintic molar ratio increased. Only systematic Δ*δ*s within the molar ratio series were considered to be caused by interactions between polyphenols and anthelmintics. Both upfield and downfield Δ*δ*s were observed; the downfield Δ*δ*s of the OHs were considered to be linked to the formation of hydrogen bonds while the upfield and downfield Δ*δ*s of the aliphatic and aromatic protons were attributed to hydrophobic interactions between the interacting compounds.^40,42,43^ Moreover, qualitative changes in the signal shapes were detected, also indicating changes in the chemical environment due to the interactions. While the systematic Δ*δ*s and qualitative changes observed for the anthelmintics could partly result from changes in the concentrations^42^ of IVM and TBZ within the molar ratio range, the comparison of these Δ*δ*s across the molar ratio series of different polyphenols still provided qualitative insights into the interactions.

### 2.1 Interactions of Flavan-3-ols and Proanthocyanidins with Ivermectin and Thiabendazole

The interactions of flavan-3-ols EC, EGC, ECG, and EGCG, and two EC based dimers, PC A2 and PC B2, with IVM and TBZ were systematically studied at different polyphenol to anthelmintic molar ratios. In general, the results showed that the flavan-3-ols and PAs mainly interacted with the anthelmintics through hydrogen bonding, as discussed in detail below. The interactions could be observed through Δ*δ*s of the protons of the interacting species but also through signal shape changes for the hydroxyl protons of the polyphenols and protons of TBZ.

In the mixtures of EC and IVM, downfield Δ*δ*s clearly occurred in the OH groups of both EC and IVM, indicating interaction (Figure S14A). Moreover, the signals of the OH groups of EC that appeared as broad peaks or humps in the ^1^H NMR spectrum of the pure EC sample, due to quick proton exchange of the hydroxyl groups and residual water in the sample, appeared as sharp peaks at all molar ratios of EC to IVM, except at the molar ratio of 3:1 (Figure 2), suggesting that the interaction between EC and IVM slows down the rapid proton exchange of the hydroxyls of EC. At the molar ratio of 3:1, the relative amount of IVM was probably not high enough to dampen proton exchange.

**Figure 2:**
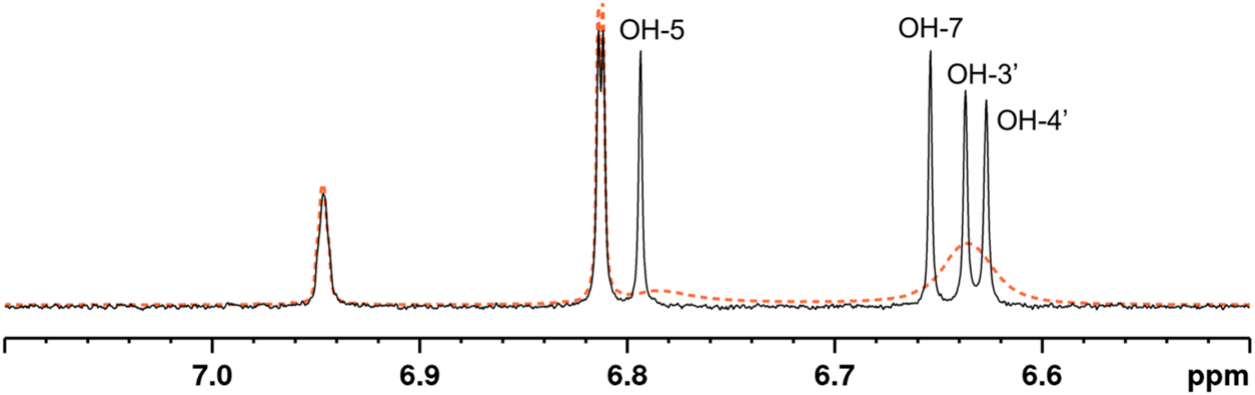
^1^H NMR spectrum showing the hydroxyl group signals of epicatechin (black line) at an epicatechin to ivermectin molar ratio of 1:1. The ^1^H NMR spectrum of the pure epicatechin sample is depicted as a dashed orange line.

In the EC-TBZ mixtures, the downfield Δ*δ*s were larger, clearer, and more systematic across EC to TBZ molar ratios for the phenolic OH groups of EC (OH-3′, OH-4′, OH-5, and OH-7) than those in the EC-IVM mixtures (Figures S14A and B). In addition, small systematical Δ*δ*s of OH-3 in the EC-TBZ mixtures were more distinct than those of the EC-IVM mixtures, suggesting that an interaction between TBZ and EC was also possibly taking place at OH-3 as the TBZ to EC ratio increased. Small but systematic Δ*δ*s of nonhydroxyl protons at H-6 and H-8 of EC were also observed in the EC-TBZ mixtures as the ratio of TBZ to EC increased. The interaction between EC and TBZ also affected the proton signals of TBZ, as evidenced by the decoalescence of the separate broad signals of H-5 and H-8 observed at higher TBZ ratios to separate broad signals as the proportion of EC increased in the sample (Figure 3). The appearance of separate signals for H-5 and H-8 of TBZ at equivalent and excess levels of EC can possibly be attributed to interaction via hydrogen bonding of EC hydroxyls to the nitrogens in TBZ, resulting in hindrance of the internal rotation of the aromatic ring moieties around the C-2–C-10 bond in TBZ.^44^ Furthermore, while no clear Δ*δ*s were observed for the broad signals of H-6 and H-7 of TBZ upon addition of TBZ, the shapes of the signals became sharper, revealing their splitting patterns and providing further proof that EC affected the internal rotation in TBZ and interacted with TBZ (Figure 3). Small systematic Δ*δ*s of H-12 in TBZ were also observed in the mixtures with EC with the increasing molar ratio of TBZ.

**Figure 3:**
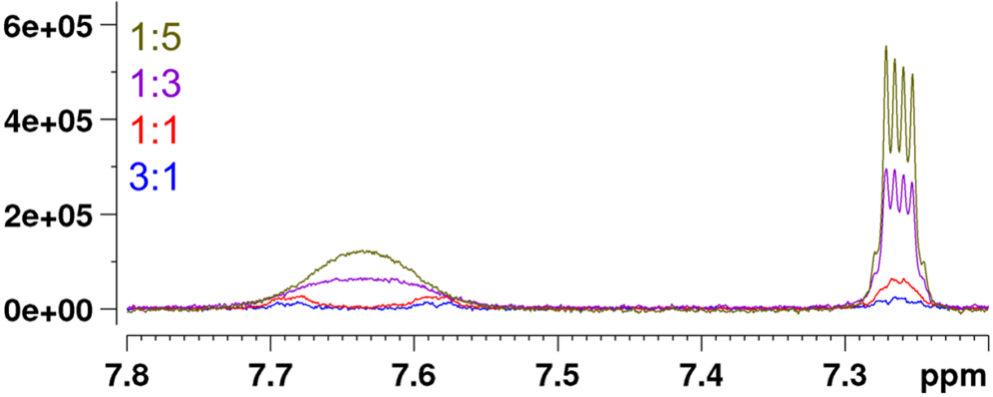
Qualitative changes in the ^1^H NMR spectra of thiabendazole (TBZ) as a result of its interaction with epicatechin. The signals of H-5 and H-8 of TBZ at 7.64 ppm decoalesced into separate broad singlets at 7.69 and 7.59 ppm with increase in epicatechin proportion.

For ECG-IVM mixtures, the largest Δ*δ*s were mainly observed for the OH groups of both compounds (Figure S15A). Similarly to EC, the broad signals of the phenolic hydroxyl groups in the NMR spectrum of ECG were converted to sharp peaks upon addition of IVM, indicating an interaction between the two compounds. Interestingly, downfield changes occurred in the chemical shifts of all the hydroxyl groups of IVM and ECG at the ECG to IVM ratios of 3:1, 1:1, and 1:3, without any further changes being observed at lower ECG to IVM molar ratios.

Unlike the ECG-IVM mixtures, Δ*δ*s were observed for nearly all protons in the ECG-TBZ mixtures throughout the ECG to TBZ molar ratio range (Figures S15A and B). The magnitudes of the Δ*δ*s were also much larger than those for the ECG-IVM mixtures. The OH-3′/OH-4′ and OH_G_-4/OH_G_-6 of ECG showed very large Δ*δ* values across all ECG to TBZ molar ratios, suggesting hydrogen bonding between the molecules via similar mechanisms as with EC and TBZ. These were the largest Δ*δ*s for the OHs of all the studied compounds, indicating strong interaction between TBZ and the catechol and galloyl units of ECG. In the ^1^H NMR spectra, the signals of OH-3′/OH-4′ of ECG overlapped in all other ECG-anthelmintic mixtures but were separated at the highest ratios of TBZ. In addition, weak but clear and systematic downfield Δ*δ*s were observed for H_G_-3 and H_G_-7 of ECG in the ECG-TBZ mixtures that were indistinguishable in the ECG-IVM mixtures. Unlike in the EC-TBZ mixtures (Figure S14B), systematic Δ*δ*s were observed for aromatic and aliphatic protons with all ECG-TBZ molar ratios, indicating that the interaction was strengthened by the galloyl group in ECG. In fact, systematic Δ*δ*s were observed for nearly all protons of ECG in the ECG-TBZ mixture, apart from H-4β and H-6. In addition, similar qualitative changes in the shapes of the H-5/H-8 and H-6/H-7 signals of TBZ were observed, as noted for the EC-TBZ mixtures, and these supported the presence of interaction via hydrogen bonding.

For the EGC-IVM mixtures, the Δ*δ*s for the phenolic hydroxyl groups of EGC were the largest among the observed signals and shifted downfield with the addition of IVM (Figure S16A). The OH-3 signal of the heterocyclic ring of EGC was clearly affected by IVM addition, but the magnitudes of Δ*δ*s were smaller than those for phenolic hydroxyls. OH-4′ of EGC exhibited larger Δ*δ*s than those of OH-5, OH-7, and OH-3′/OH-5′. The effects of IVM addition on the Δ*δ*s of other than hydroxyl protons were minimal.

For EGC-TBZ mixtures, the downfield Δ*δ*s of the hydroxyl groups of EGC were larger than those in EGC-IVM mixtures (Figure S16B). Here as well, OH-4′ showed the largest Δ*δ*s and the Δ*δ*s of OH-3′/OH-5′ were also slightly larger than those of OH-5 and OH-7. OH-3 of the heterocyclic ring also exhibited distinct systematic downfield changes. Small systematic Δ*δ*s were observed for H-6 and H-8 of EGC, with downfield changes observed for H-6 and upfield changes for H-8. Systematic Δ*δ*s were observed for H-12 and H-5/H-8 of TBZ, although they were quite small. Qualitatively, similar shape changes for the signals H-5/H-8 and H-6/H-7 of TBZ were observed in EGC-TBZ mixtures as noted above in EC-TBZ and ECG-TBZ mixtures, also suggesting hydrogen bonding via similar mechanisms as previously discussed.

In EGCG-IVM mixtures, the proton signals of OH groups in EGCG were affected more than those of the other protons (Figure S17A). Small systematic changes were, however, observed for H-4α and H-6 of EGCG with downfield and upfield Δ*δ*s, respectively. Similarly to the effect of IVM on the Δ*δ*s of ECG, the Δ*δ*s of OH groups in EGCG were similar at molar ratios of 1:3 and 1:5, but were higher at a molar ratio of 1:10. The magnitudes of Δ*δ*s of OH-5 and OH-7/OH_G_-5 of EGCG were similar throughout the molar ratio range, while the Δ*δ*s of OH-4′, OH-3′/OH-5′ and OH_G_-4/OH_G_-6 fluctuated throughout the molar ratio range.

In EGCG-TBZ mixtures, the Δ*δ*s of EGCG were larger than those observed in EGCG- IVM mixtures. The magnitudes of the downfield Δ*δ*s of OH-4′, OH-5, and OH-7/OH_G_-5 in EGCG were similar (Figure S17B). The largest downfield Δ*δ*s were observed for OH-3′/OH-5′, and the magnitudes of the downfield Δ*δ*s for OH_G_-4/OH_G_-6 were only slightly smaller. Interestingly, clear and systematic upfield Δ*δ*s were observed for H-8 of EGCG, while almost no changes in Δ*δ*s were observed for H-6 as the molar ratio of TBZ gradually increased (Figure 4). Small upfield Δ*δ*s were also observed for H-4β and downfield shifts for H-2′/H-6’ and H_G_-3/H_G_-7. Qualitatively, similar distinct changes in the peak shapes of H-5/H-8 and H-6/H-7 of TBZ were also observed, as noted for the other flavan-3-ols, indicating hydrogen bonding between the molecules via similar mechanisms as described with other flavan-3-ols.

**Figure 4:**
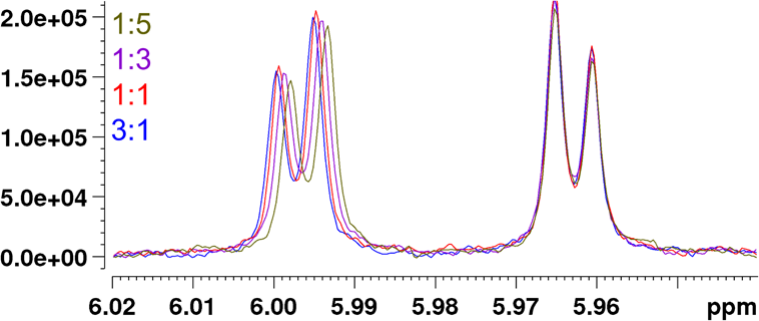
Partial ^1^H NMR spectra of epigallocatechin gallate (EGCG)-thiabendazole (TBZ) mixtures. Systematic upfield change in the chemical shift of H-8 in EGCG at about 5.997 ppm was observed as the molar ratio of TBZ increased, whereas practically no change in the chemical shift was observed for H-6 of EGCG at 5.963 ppm (Figures 1 and S17B).

The degree of hydroxylation of the B-ring of the flavan-3-ols was noted to impact interactions between flavan-3-ols and anthelmintics. For example, in EC- and EGC-IVM mixtures, the Δ*δ*s of the phenolic OHs of EGC were distinctively larger and more systematic than those of EC (Figures S14A and S16A). These differences are most likely due to differences in the interaction strengths between these flavan-3-ols and the anthelmintics, since IVM can form more hydrogen bonds with EGC than with EC due to an extra OH in EGC, In EC- and EGC-TBZ mixtures, the magnitudes of Δ*δ*s of the phenolic OHs of EC and EGC were mostly similar, apart from those of OH-4′ of EGC (Figures S14B and S16B) which were much larger, indicating stronger interaction. Furthermore, differences between the interactions with TBZ and the galloylated flavan-3-ols were observed, as when the effects of TBZ on the Δ*δ*s of ECG and EGCG were examined, the Δ*δ*s of ECG were generally larger than those of EGCG after TBZ addition. This was the case especially for the OH_G_-4/OH_G_-6 of ECG, whose Δ*δ*s became larger than for those of EGCG as the molar ratio of TBZ increased (Figures S15B and S17B). Although clear differences in the Δ*δ*s of many of the signals of different flavan-3-ols were observed in the polyphenol-anthelmintic mixtures, the Δ*δ*s of selected signals of IVM and TBZ, i.e., those that showed clear systematic Δ*δ*s or other distinct qualitative changes, were relatively similar (Figure 5).

**Figure 5:**
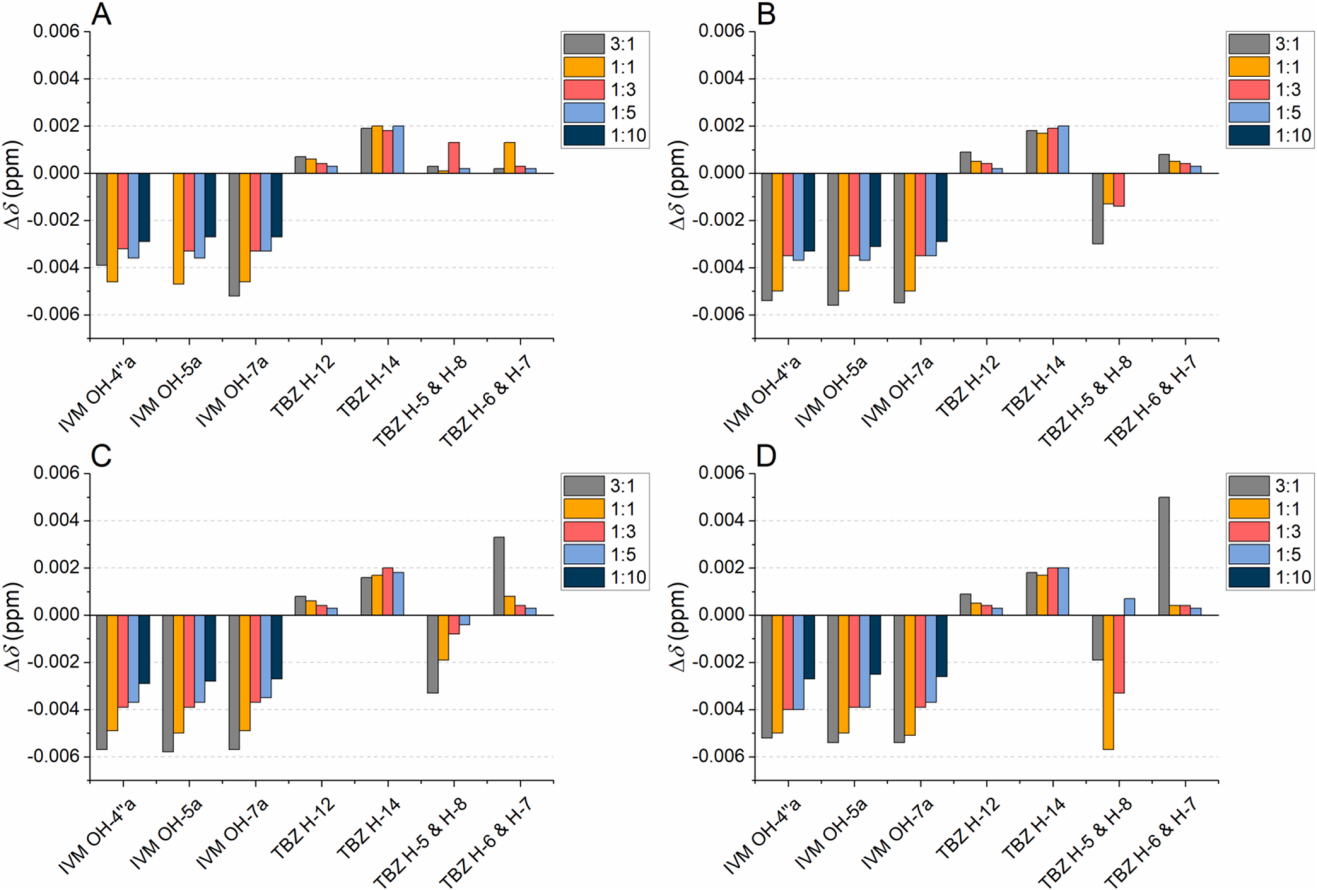
Chemical shift changes (Δ*δ*s, Δ*δ* = *δ*_mixture_ – *δ*_pure compound_) of selected signals of ivermectin (IVM) and thiabendazole (TBZ), i.e., signals with clear systematic Δ*δ*s or other qualitative changes, as a function of decreasing flavan-3-ol to anthelmintic molar ratio: (A) epicatechin (EC), (B) epicatechin gallate (ECG), (C) epigallocatechin (EGC), and (D) epigallocatechin gallate (EGCG).

In the ^1^H NMR spectra of the PC dimer A2-IVM mixtures, the single broad OH signal in the spectrum of the pure PC A2 sample split into separate sharper signals after the addition of IVM. The absolute Δ*δ*s of the signals of the OH groups of PC A2 in the mixtures were large because the *δ* of the original broad OH signal of the pure sample was used as the base value for calculating the Δ*δ*s for all the separated OH signals (Figure S18A). However, the relative Δ*δ*s for each OH signal within the molar ratio series were small but systematic. Interestingly, larger Δ*δ*s were observed for the OH-3 of the extension unit of PC A2 than for that of EC, while the Δ*δ*s of OH-3b in the terminal unit of the PC dimer A2 were similar to those in the PC monomer. Other differences in the magnitudes of the Δ*δ*s of the OHs were found between the extension and terminal units of PC A2 as well, as clearly stronger interaction, i.e., larger Δ*δ*s, were observed for OH-5b in the terminal unit than for OH-5 in the extension unit. While no large Δ*δ*s were observed for the other protons of PC A2, besides those in the hydroxyl groups, small Δ*δ*s were observed across the molar ratio range for H-3 and H-3b, as well as very small changes for H-2′, H-2b, H-2′b, H-6, and H-6’.

In the PC A2-TBZ mixtures, the observed Δ*δ*s of PC A2 were broadly similar to those of PC A2-IVM mixtures, as small Δ*δ*s were also observed for the PC A2-TBZ mixtures. However, the magnitudes of the Δ*δ*s were larger than those for the PC A2-IVM mixtures. The OH-3 and OH-3b of PC A2 in the PC A2-TBZ mixtures showed clear systematic Δ*δ*s as a function of molar ratio, with the OH-3 showing larger Δ*δ*s than OH-3b, while OH-3b showed comparable Δ*δ*s to those observed for OH-3 of EC in the EC-TBZ mixture (Figures S14B and S18B). Small but systematic upfield Δ*δ*s were observed for H-2b, H-3, H-3b, H-6’, H-6b, and H-8 of PC A2, as well as small but systematic downfield Δ*δ*s for H-2′ and H-2′b. Slightly larger Δ*δ*s were observed for H-2′b in the terminal unit than for H-2′ in the extension unit, but more distinguishable differences between these two units were observed for the hydroxyl groups. In PC A2-TBZ mixtures, the differences in the Δ*δ*s of the OHs between the extension and the terminal unit were more pronounced than in PC A2-IVM mixtures since the Δ*δ*s were slightly larger for the signals of OH-3, OH-3′, and OH-4′ groups in the extension unit than those in the terminal unit. By contrast, the Δ*δ*s of OH-5b of the terminal unit were larger than for OH-5 of the extension unit. Qualitatively, the shape changes of the signals of TBZ in PC A2-TBZ mixtures were similar to those in the other previously studied mixtures, i.e., the separate signals of H-5 and H-8 of TBZ coalesced into a single broad signal and the signal for H-6/H-7 became sharper with the increase in the molar ratio of TBZ.

In PC B2-anthelmintic mixtures, the results could not be directly compared with those of other mixtures, because the interactions of this compound could mainly be studied at 243 K and the noncovalent interactions of polyphenols have previously shown temperature dependence.^45^ At 298 K, only the signal for OH-3 of PC B2 could be characterized with certainty as the spectrum measured at 298 K is affected by the hindered rotation around the C-4–C-8 bond of PC B2 that results in uncharacterizable broad peaks.^46^ In the spectrum measured at 243 K, two sets of signals of PC B2 were observed, representing two different conformers, of which the major conformer was investigated for Δ*δ*s.^47,48^ From the spectra measured at 298 K, clear systematic Δ*δ*s in the aromatic region of the ^1^H NMR spectrum of PC B2 were observed, likely corresponding to the hydroxyl groups of the PA. For both anthelmintics at both experimental temperatures, systematic Δ*δ*s were also observed for the OH-3 of PC B2, with larger Δ*δ*s observed with PC B2-TBZ mixtures than with PC B2-IVM mixtures (Figures S19 and S20). At 298 K, the Δ*δ*s for the selected signals of the anthelmintics in PC A2- and PC B2-IVM mixtures and PC A2- and PC B2-TBZ mixtures were similar, but the Δ*δ*s of the anthelmintics in PC B2-anthelmintics mixtures at 243 K differed, especially for the PC B2-IVM mixtures (Figure 6), likely as the *δ*s of OHs are sensible to temperature and concentration and because the noncovalent interactions of polyphenols have been shown to be temperature dependent.^42,45^ In PC B2-TBZ mixtures, at both 298 and 243 K, similar qualitative peak shape changes of the signals of TBZ were observed as noted for previously studied TBZ mixtures, although at 243 K, the H-5 and H-8 signals of pure TBZ were two sharp doublets that turned into one broad singlet upon interaction with PC B2. The sharp signals of H-5 and H-8 of TBZ at 243 K support the notion of hindered rotation of the C-2–C-10 of TBZ due to interaction with polyphenols, as further discussed above, regarding the interactions with EC.

**Figure 6:**
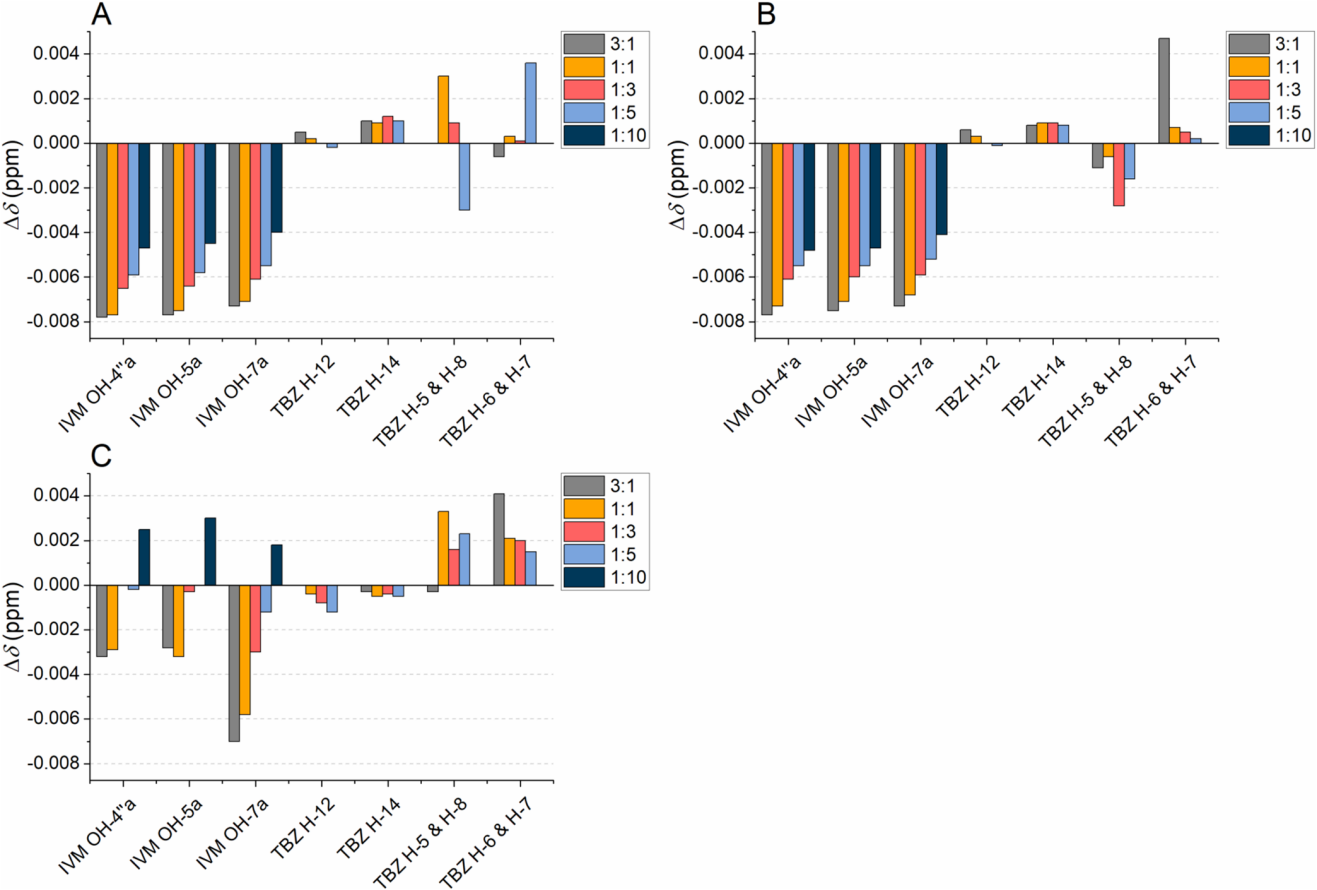
Chemical shift changes (Δ*δ*s, Δ*δ* = *δ*_mixture_ – *δ*_pure compound_) of selected signals of ivermectin (IVM) and thiabendazole (TBZ) in the ^1^H NMR spectra at different molar ratios of proanthocyanidins to anthelmintics: (A) procyanidin A2, (B) procyanidin B2 at 298 K, and (C) procyanidin B2 at 243 K.

Similarly as observed for flavan-3-ols and PC A2 at 298 K, the hydroxyl groups of PC B2 were strongly involved in interactions with IVM and TBZ at 243 K. Differences in the Δ*δ*s of the OHs of PC B2 in PC B2-IVM and PC B2-TBZ mixtures were observed. In the case of IVM, the magnitudes of Δ*δ*s were much more similar between phenolic and nonphenolic OHs, while in the case of TBZ, the phenolic OHs exhibited much larger Δ*δ*s than those of nonphenolic OHs as a function of increasing molar ratio of TBZ. The OHs exhibiting the largest Δ*δ*s differed depending on the anthelmintic in the mixture as with IVM the largest Δ*δ*s within the molar ratio range were observed for OH-7 of PC B2, whereas OH-5 and OH-5b exhibited similar but smaller Δ*δ*s. In the spectra of PC B2-TBZ mixtures, OH-5 and OH-7b of PC B2 clearly exhibited the largest Δ*δ*s. Interestingly, O’Kennedy et al. reported that, determined by low temperature NMR experiments, the two conformers of PC B2 in equilibrium with each other in acetonitrile are partially compact (major) and the fully extended (minor) conformers, and in both conformers, OH-5 and OH-7b are spatially close to each other.^49^

In PC B2-IVM mixtures at 243 K, systematic Δ*δ*s of nonhydroxyl protons were observed only for H-2b and H-3b of PC B2, while in PC B2-TBZ mixtures, systematic changes were observed for nearly all characterized protons: H-2, H-2b, H-3, H-4, H-6, H-6b, and H-8 (Figure S19), indicating stronger interaction with TBZ than with IVM. In PC B2-TBZ mixtures, the Δ*δ*s for H-2b and H-8 were upfield, while the others were downfield. This is different than what was observed for PC A2 or the flavan-3-ols, where most of the Δ*δ*s of nonhydroxyl protons were upfield. Clear differences in the magnitudes of the Δ*δ*s between the extension and the terminal units were observed for H-2 and H-2b and for H-3 and H-3b, with the largest Δ*δ*s observed for the protons of the extension unit. However, H-6 of the extension unit and H-6b of the terminal unit of PC B2 exhibited similar magnitudes of Δ*δ*s in the presence of TBZ.

Overall, the results showed that Δ*δ*s occurred when these polyphenols were mixed one to one with the two anthelmintics, indicating possible interaction. The interactions of these polyphenols with both commercial anthelmintics relied heavily on but were not limited to the hydroxyl groups of the polyphenols. The interaction sites of IVM exhibited less variability than those of the polyphenols, because the hydroxyl groups of IVM were the sole interacting sites between IVM and polyphenols. The Δ*δ*s of the OH groups of IVM could also be affected by changes in the concentrations^42^ but the Δ*δ*s across the molar ratio series could be compared between the different polyphenols. Qualitative changes in the shapes of H-5/H-8 and H-6/H-7 signals of the benzo-moiety in TBZ (Figure 2) upon interaction with polyphenols were observed for all polyphenols studied, indicating that the environment of these protons was affected by the presence of the polyphenol in the mixture, suggesting hydrogen bonding between the molecules. The presence of hydrogen bonding in the interactions with TBZ is also supported by the large systematic Δ*δ*s observed for the OH groups of the flavan-3-ols and dimeric PAs.

### 2.2 Interactions of Hydrolyzable Tannins with Ivermectin and Thiabendazole

The same molar ratios as those used for studying flavan-3-ols and PA dimers were used to study the interactions between the two anthelmintics and HTs. The interactions of IVM with HTs were different from those of flavan-3-ols with IVM, as for most of the HTs studied, the presence of IVM induced small and systematic changes in the chemical shifts of protons throughout the HT structure. In general, larger magnitudes of Δ*δ*s were recorded for the interactions between HTs and TBZ than for those between HTs and IVM. Qualitative changes in the shapes of the signals of H-6/H-7 and H-5/H-8 of TBZ were similar to those observed for flavan-3-ols and PA dimers but were more distinct, occurring at lower molar ratios of the anthelmintic. Additionally, qualitative signal shape changes were also observed for IVM protons in the interactions between HTs and IVM, which were not observed in the interactions between IVM and flavan-3-ols or dimeric PAs (Figure 7). These changes mainly corresponded to broadening of the peaks for H-4″, OH-4″a, OH-5a, and OH-7a of IVM (Figure 1, Figure S2). These are hydroxyl groups in different parts of the IVM molecule except for H-4″ being the proton of the adjacent carbon of OH-4″a, located in the disaccharide moiety of IVM. Furthermore, the hydroxyl group of the ethanol impurity of IVM at 2.44 ppm was also affected by the broadening effect of HTs. These line broadening effects could indicate of hydrogen bonding by these hydroxyl groups, supported by the fact that at the same time other proton signals of IVM did not show any line broadening. Furthermore, the OH-5a and OH-7a signals experienced clear systematic downfield Δ*δ*s as a function of molar ratio, giving further proof of possible hydrogen bonding by these hydroxyls.

**Figure 7:**
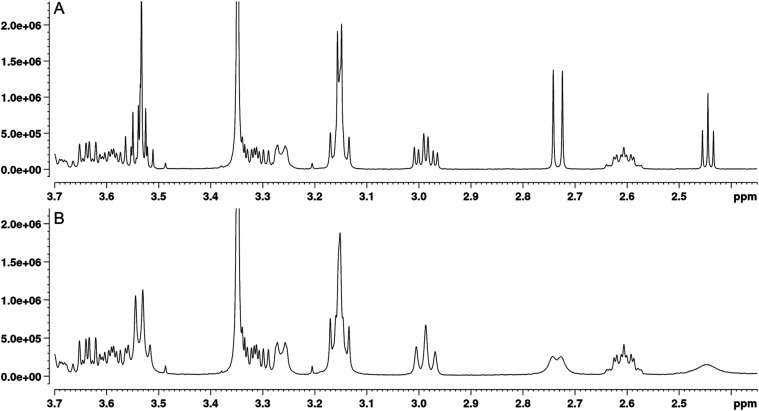
Examples of the broadening of the proton signals of ivermectin (IVM) and ethanol visible at *δ*s of 2.44 ppm (ethanol OH impurity), 2.73 ppm (OH-5a), 2.98 ppm (H-4″), 3.15 ppm (OH-4″a, H-2, H-4′), and 3.53 ppm (OH-7a, ethanol CH_2_ impurity). (A) ^1^H NMR spectrum of pure IVM and (B) ^1^H NMR spectrum of a tellimagrandin II-IVM mixture at a tellimagrandin II to IVM molar ratio of 1:10.

Tellimagrandin I exists in equilibrium of α and β anomers with respect to the anomeric position at C-1. The configuration of the anomeric position affected the interaction between the tannin and IVM, as different trends were observed in the Δ*δ*s of various signals of the different anomers (Figure S21A). Generally, the Δ*δ*s for tellimagrandin I and IVM stayed mostly at even levels across the molar ratio range, but systematic upfield trends within the molar ratio range were observed for H_C_-3α, H_C_-3β, H-3α (but not for H-3β), and H-1β (but not for H-1α) of tellimagrandin I. All possible Δ*δ*s could not be observed because IVM exhibited overlapping signals with tellimagrandin I, especially with H-2α and H-2β. Larger downfield effects within the molar ratio range were observed for the OH-5a and OH-7a of IVM. Qualitatively, a broadening effect was observed for the H-4″ signal of IVM and signal shape changes in the OHs were present, similar to those depicted in Figure 7. The differences in Δ*δ*s observed between α and β anomers of tellimagrandin I, e.g., larger Δ*δ*s for H-3α than for H-3β and larger Δ*δ*s for H-1β than for H-1α, could be linked to hydrogen bonding of IVM to tellimagrandin I from different directions. The axial OH-1α in the α-anomer seems to direct IVM to approach tellimagrandin I axially, indicated by the upfield Δ*δ*s of the axial H-3α, but practically no effect was observed with the equatorial H-1α or the axial H-4α on the opposite side of the ring, whereas with the β-anomer the equatorial OH-1β directs IVM to approach tellimagrandin I from equatorial direction that is reflected in the upfield Δ*δ*s of the axial H-1β.

In tellimagrandin I-TBZ mixtures, larger magnitudes of Δ*δ*s were observed than in tellimagrandin I-IVM mixtures, and Δ*δ*s within the molar ratio range were observed for substantially higher number of proton signals (Figures S21A and B). Also, in tellimagrandin I-TBZ mixtures, differences in the Δ*δ*s were observed between the two anomers of tellimagrandin I. These differences were distinct for some protons of tellimagrandin I as only small downfield Δ*δ*s within the molar ratio range were observed for H-1α and H-5α, whereas large upfield changes were observed for H-1β and H-5β with the increase of the molar ratio of TBZ. H-3β also exhibited larger upfield Δ*δ*s than its counterpart in the α-anomer, suggesting that the orientation of the equatorial OH-1β would be more favorable for interactions with TBZ than the axial OH-1α. H-2α, located axially on the other side of the ring than H-1β, H-5β, and H-3β, exhibited slightly larger upfield Δ*δ*s than its anomeric counterpart. Although differences were observed in the glucose protons, the configuration of the anomeric position in tellimagrandin I had little impact on the interaction susceptibilities of the galloyl groups and the HHDP group, since the Δ*δ*s for these protons were similar between the anomers (Figure S21B). The Δ*δ*s of tellimagrandin I proton signals were mainly upfield but those of H_D_-3α and H_D_-3β were slightly downfield. This could indicate that these protons are oriented differently in regard of the aromatic rings of TBZ due to shielding and deshielding ring-current effects of the aromatic moieties of TBZ.^43,50^ As the Δ*δ*s for H_C_-3α and H_C_-3β were the largest and clearly upfield, the orientation of the aromatic structures of TBZ might be parallel to the ring C of tellimagrandin I (Figure 1), leading to H_D_-3α and H_D_-3β experiencing a deshielding ring-current effect (Figure S21B).^37,41^ Small systematic upfield Δ*δ*s were also observed for TBZ protons H-14 and H-6/H-7, although these changes were much smaller than those of tellimagrandin I. In addition to the possible hydrophobic interactions, similar types of qualitative changes in the signal shape and coalescence were observed for H-6/H-7 and H-5/H-8 of TBZ, as observed above for PAs and flavan-3-ols, but with tellimagrandin I, the changes were more distinct, indicating stronger interaction via hydrogen bonding (Figure 2).

The observed Δ*δ*s in tellimagrandin II-IVM mixtures were relatively small compared with those in tellimagrandin I-IVM mixtures, indicating smaller degree of interaction. Similar magnitudes of Δ*δ*s were observed for H-1 of tellimagrandin II as for H-1β of tellimagrandin I (Figures S21A and S22A). The largest Δ*δ*s within the molar ratio range were observed for OH-5a of IVM while minimal changes were observed for protons of tellimagrandin II other than for H-1. However, qualitative changes indicating interaction, were observed for proton signals of tellimagrandin II. In the ^1^H NMR spectrum of pure tellimagrandin II, the signals for H_B_-3/H_B_-7 and H_C_-3/H_C_-7 overlap but in tellimagrandin II-IVM mixtures they are clearly separate, although no Δ*δ*s were observed across the tellimagrandin I to IVM molar ratio range (Figure 8).

**Figure 8:**
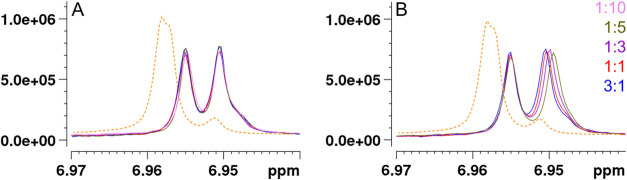
Signals H_B_-3/H_B_-7 and H_C_-3/H_C_-7 of tellimagrandin II (Figure 1) overlap in the ^1^H NMR spectrum of pure tellimagrandin II (orange dashed line) but separate into individual characterizable signals in mixtures of tellimagrandin II and (A) ivermectin and (B) thiabendazole (TBZ). In the interaction with TBZ (B), H_B_-3/H_B_-7 of tellimagrandin II exhibits upfield chemical shift changes with the increase in the molar ratio of TBZ.

The Δ*δ*s observed in tellimagrandin II-TBZ mixtures were more numerous, and the overall Δ*δ*s were more distinct than those in the tellimagrandin II-IVM mixtures, and systematic changes within the molar ratio range were observed in most cases (Figure S22B). These changes were observed for all protons of tellimagrandin II except for H_C_-3/H_C_-7. Similarly to the observations made using pure tellimagrandin II and IVM, the signals for H_B_-3/H_B_-7 and H_C_-3/H_C_-7 of tellimagrandin II overlap but in tellimagrandin II-TBZ mixtures, the signals separate with upfield shift changes observed for H_B_-3/H_B_-7, which is in contrast to the absence of shift changes in tellimagrandin II-IVM mixtures (Figure 8). The Δ*δ*s of H_E_-3 of tellimagrandin II were downfield, similarly to what was observed for H_D_-3 of tellimagrandin I. Although differences between the *δ*s of the pure TBZ signals and those of TBZ in tellimagrandin II-TBZ mixtures were observed, the resulting Δ*δ*s were relatively small and did not show significant systematicity with the increasing molar ratio of TBZ. In general, the observed—mainly upfield effects with the tellimagrandin II-TBZ mixtures indicate rather hydrophobic interaction between the molecules rather than hydrophilic interaction.

In the pentagalloylglucose (PGG)-IVM mixtures, the Δ*δ*s were small, and differed from those of tellimagrandins I and II, as small but systematic upfield Δ*δ*s were observed for the glucose protons of PGG: H-1, H-2, H-3, H-4, H-5, and H-6 (Figure S23A). Differences compared to tellimagrandins I and II were also noted for the downfield Δ*δ*s of the hydroxyl groups of IVM, which were most prominent at the PGG to IVM molar ratio of 3:1 (Figure S23A). The observed effects indicate rather hydrophilic interactions via hydrogen bonding between the molecules rather than hydrophobic interactions. However, the broadening of the proton signals of IVM in the presence of PGG was minimal when compared with that of the proton signals of IVM in the presence of other HTs.

In the PGG-TBZ mixtures, however, the Δ*δ*s were more pronounced, with systematic Δ*δ*s observed for both TBZ and PGG protons (Figure S23B). For TBZ, the largest Δ*δ*s were observed for H-14 and H-5/H-8 signals, while small changes were observed also for H-12, and H-6/H-7 signals. Similarly to what was observed in PGG-IVM mixtures, the most affected protons of PGG in PGG-TBZ mixtures were those in the glucose core, but unlike those in PGG-IVM mixtures, the glucose protons of PGG in the PGG-TBZ mixtures showed much larger upfield Δ*δ*s. For the galloyl groups, weaker systematic upfield Δ*δ*s within the molar ratio range were observed. As before, qualitative changes were also observed for the TBZ signals in PGG-TBZ mixtures, as shown by the coalesce of H-5 and H-8 signals into one broad signal as the proportion of TBZ was increased (as observed for TBZ in the presence of other tannins as well) and the sharpening of the H-6/H-7 signal, though this was visible only at equivalent and excess proportions of TBZ, suggesting that the presence of PGG affects the intramolecular dynamics of TBZ differently.

In vescalagin-IVM mixtures, no large gradual Δ*δ*s were observed within the molar ratio range, indicating that the interaction between IVM and this HT was weak (Figure S24A). Increase in the molar ratio of IVM to vescalagin shifted the OH-7a and OH-5a signals of IVM downfield along with minor downfield changes for H-2 and H-6 of vescalagin. Although, the Δ*δ*s of the proton signals of vescalagin observed for the vescalagin-IVM mixtures were the largest among all HTs tested, the changes with respect to molar ratio were small. Despite the weak interaction suggested by the small magnitudes of the Δ*δ*s of vescalagin with respect to molar ratio, broadening of IVM hydroxyl signals was still observed, suggesting interaction through hydrogen bonding.

For vescalagin-TBZ mixtures, systematic Δ*δ*s were observed across the molar ratio range for nearly all protons of vescalagin. Interestingly, glucose protons H-1, H-3, H-4, and H-6 exhibited downfield changes (Figure S24B) with the increase of the molar proportion of TBZ. Among the aromatic protons, H_C_-3 and H_E_-3 showed larger Δ*δ*s within the molar ratio range than H_D_-3. All Δ*δ*s associated with the aromatic protons were upfield. Clear shifts and variation within the molar ratio range were also observed for H-14 and H-6/H-7 of TBZ. Additionally, the qualitative changes in TBZ signals were similar as observed in other HT-TBZ mixtures. Notably, the H-6/H-7 signals of TBZ sharpened even at a vescalagin to TBZ molar ratio of 3:1.

For gemin A-IVM mixtures, although distinct Δ*δ*s for gemin A were observed, systematic Δ*δ*s with increasing molar ratio of IVM were relatively small, thus indicating fairly weak interaction between gemin A and IVM. Clear systematic changes within the molar ratio range were observed for the signals of OH-7a and OH-5a of IVM, whereas smaller changes were noted for H-1a, H-1b, H-2a, H-3a, H-4b, H_D_-3/H_H_-3, H_G_-3, and H_I_-3 of gemin A (Figure S25A). The chemical shifts of gemin A protons were mainly upfield with increasing molar ratios of IVM, except for H-1b and H_G_-3. Unfortunately, IVM signals overlapped with some signals of gemin A in the ^1^H NMR spectra, hampering detailed analysis in certain structural regions of gemin A. Some differences, however, were observed between the monomeric constituents of gemin A, as shown by the slightly higher Δ*δ*s for H-1b than for H-1a within the molar ratio range. The directions of the Δ*δ*s for H-1a and H-1b also differed, with H-1a showing upfield shift changes and H-1b downfield changes. Also, H-3a of gemin A exhibited small upfield changes while none were observed for H-3b.

Among the HTs studied, the gemin A-TBZ interaction was the strongest indicated by nearly 0.015 ppm Δ*δ*s observed for some gemin A protons (Figure S25B). Notably, H-14 of TBZ, as well as H-5/H-8 and H-6/H-7, showed large upfield Δ*δ*s with increasing TBZ ratios. Almost all protons of gemin A showed systematic Δ*δ*s, which were generally upfield, except for H-1b, H_A_-3, and H_G_-3. Generally, the changes associated with the b-moiety of the dimer (potentillin moiety) were smaller in magnitude than those for the a-moiety (tellimagrandin II moiety). The stronger interaction between gemin A and TBZ was also apparent in the qualitative changes in the signals of TBZ; instead of the coalesced broad signal of H-5/H-8 of TBZ observed with all other HTs, TBZ in the presence of gemin A showed sharp signals for H-5/H-8 as well as sharp signals with upfield shifts for H-6/H-7 with the increase in the molar ratio of TBZ. Mostly, the aforementioned observations of mainly upfield changes suggest rather hydrophobic interactions between the molecules than hydrophilic ones.

The comparison of the Δ*δ* profiles of selected signals of the anthelmintics, i.e., those that underwent qualitative signal shape changes or those for which clear Δ*δ*s were observed, revealed more variability in the Δ*δ*s of anthelmintics in the presence of HTs (Figure 9) than in the presence of flavan-3-ols and PAs (Figures 5 and 6). Among IVM signals, the profiles of the OH-4″a signals were similar across most of the IVM-HT mole ratio series and they exhibited no systematic shift changes despite the clear changes in the signal shapes. This disaccharide end of the IVM molecule seems to be less important in the interactions with HTs. Small systematic downfield Δ*δ*s for OH-4″a were only observed in interactions between PGG and IVM.

**Figure 9:**
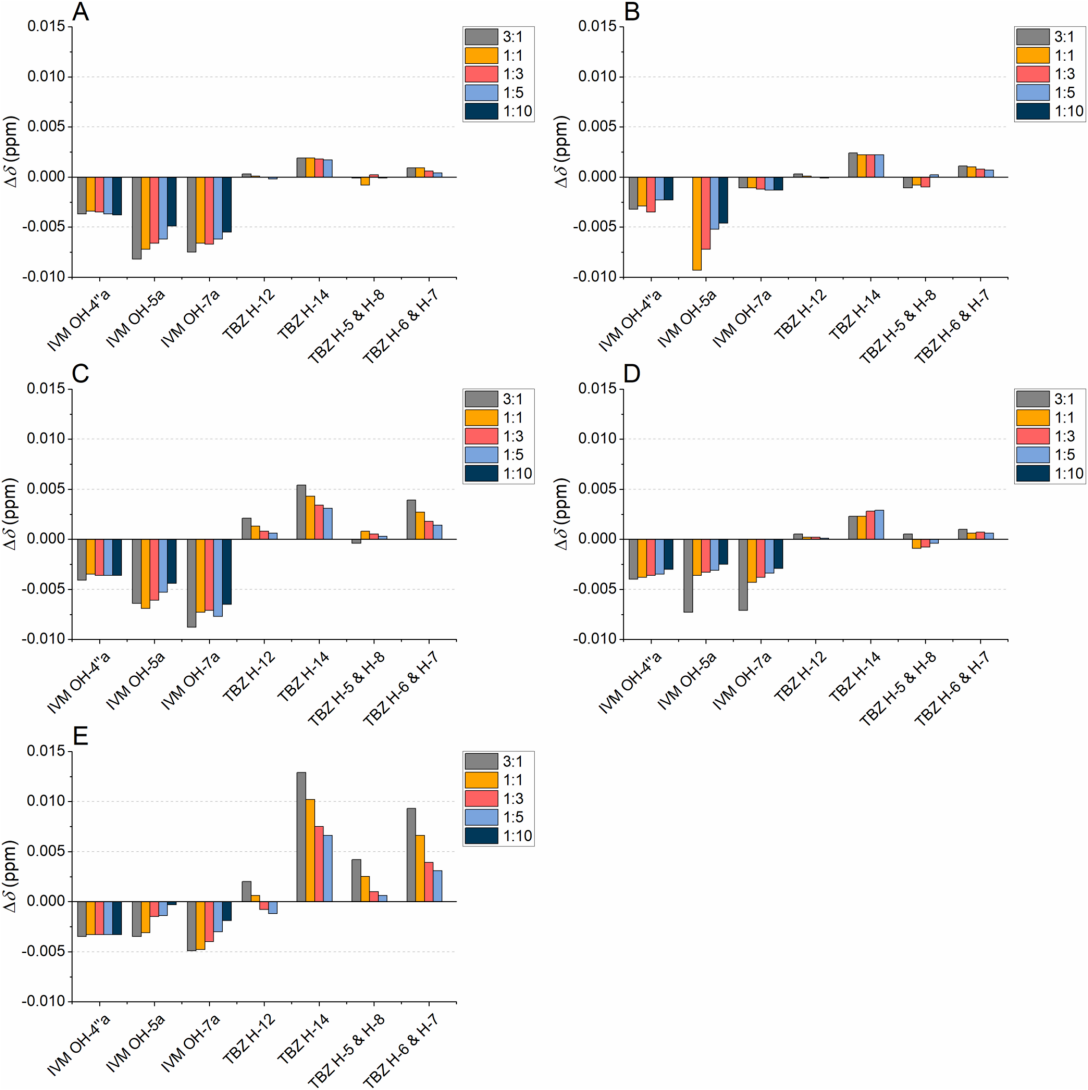
Chemical shift changes (Δ*δ*s, Δ*δ* = *δ*_mixture_ – *δ*_pure compound_) of selected signals in ^1^H NMR spectra of ivermectin (IVM) and thiabendazole (TBZ) at different molar ratios of hydrolyzable tannins to anthelmintics: (A) tellimagrandin I, (B) tellimagrandin II, (C) vescalagin, (D) pentagalloylglucose, and (E) gemin A.

Among other IVM signals, the Δ*δ* profiles of OH-5a and OH-7a of IVM exhibited similar profiles to each other in interactions between IVM and all other model HTs except for tellimagrandin II. Contrary to the trends of other model HTs, in interactions between tellimagrandin II and IVM, OH-7a of IVM showed no Δ*δ*s with respect to molar ratio while OH-5a showed the largest Δ*δ*s within the HT molar ratio range. These differences, combined with the systematic structural differences between tellimagrandin I, tellimagrandin II, and PGG, throw light on the effects of galloyl groups and HHDP groups on the interaction mechanisms of IVM with HTs. The additional galloyl group contributes to the higher Δ*δ* of OH-5a of IVM in the tellimagrandin II-IVM interaction than in the tellimagrandin I-IVM interaction, while seeming to simultaneously hinder the interaction of the OH-7a of IVM with tellimagrandin II. This obstruction is probably linked to the rather rigid HHDP group of tellimagrandin II, since it was not observed in the interactions between IVM and structurally more flexible PGG and IVM (Figure 9).

In the presence of TBZ, tellimagrandin I, tellimagrandin II, and PGG exhibited similar Δ*δ* profiles whereas vescalagin and gemin A exhibited different profiles to those, and the interaction of TBZ with gemin A resulted in systematic Δ*δ*s for all TBZ protons inspected (Figure 9). Dimeric gemin A has fewer freely rotating galloyl groups than monomeric tellimagrandin II or PGG, but has multiple HHDP groups instead, which most likely contributed to the interaction between gemin A and TBZ, because due to its small size, TBZ is less unlikely affected by differences in the flexibilities of galloyl and HHDP groups. Interestingly, in our previous studies of TBZ-gemin A interactions using ITC, gemin A exhibited similar enthalpy changes to those of PGG, reflecting equal interaction strengths for gemin A and PGG.^33^ Here, however, larger Δ*δ*s were observed for gemin A than for PGG. These differences, however, could arise from the different molar ratios of the compounds and experimental setups between these studies.

### 2.3 Differences in Interaction Mechanisms of the Two Anthelmintics

The two anthelmintics are representatives of two different anthelmintic classes (IVM for macrocyclic lactones, TBZ for benzimidazoles), and as expected, demonstrated different interaction mechanisms with model polyphenols. The presence of TBZ affected the chemical shifts of the aromatic and nonaromatic protons of the studied polyphenols and both upfield and downfield Δ*δ*s were observed with increases in the molar ratio of TBZ. Clear differences in the interaction mechanisms were also observed depending on the polyphenol class. For example, the Δ*δ*s and the qualitative changes of the signals of the aromatic protons of TBZ were more distinct with HTs than with flavan-3-ols or dimeric PAs. These differences are likely due to the presence of galloyl moieties in HT structures, which can form hydrophobic interactions with the aromatic rings of TBZ in addition to hydrogen bonds. The largest Δ*δ*s for the signals of polyphenols in the interactions with TBZ were, however, observed for the OH groups of flavan-3-ols.

The Δ*δ* profiles of the selected signals of IVM varied depending on the class of the polyphenol (Figures 5, 6, and 9), illustrating the different interaction mechanisms between model polyphenols and IVM. In the case of flavan-3-ols and PAs, the Δ*δ* profiles of OH-4″a, OH-5a, and OH-7a of IVM were generally similar to each other but in the case of HTs, the OH-4″a generally exhibited smaller Δ*δ*s within the molar ratio range than those of other OHs of IVM, indicating that the disaccharide end of the IVM molecule is less important in the interactions of IVM with HTs. While hydrogen bonding was evident between the OHs of IVM and flavan-3-ols and PAs, IVM seemed to induce more Δ*δ*s in the aliphatic and aromatic protons of HTs than in those of flavan-3-ols or PAs indicating that also hydrophobic interaction mechanisms may contribute to the overall interactions between HTs and IVM. The different interaction mechanisms involved were further confirmed by the observed qualitative changes in IVM signals, which were only detected in IVM interactions with HTs.

In our previous studies on interactions between flavan-3-ols, PAs, and HTs with TBZ, increased enthalpy change, corresponding to increased interaction strength, was directly linked to the presence of freely rotating galloyl groups and increased molecular weight.^33,34^ Here, similar trends were observed, as shown by the link between the Δ*δ*s and these structural characteristics, i.e., the number of aromatic OHs and galloyl groups, and degree of polymerization. With the increasing TBZ molar ratio in polyphenol-TBZ mixtures, the Δ*δ*s for aliphatic and aromatic protons of tannins were mainly upfield, indicating shielding ring-current effects deriving from the hydrophobic interactions of the aromatic rings, with the assumption that the shielding effect also extends to the aliphatic protons.^43,50^ However, there were exceptions for all polyphenol classes, as evidenced by, e.g., the downfield shifts of H-1b, H_A_-3, and H_G_-3 of gemin A upon its interaction with TBZ. As the direction of the Δ*δ* can reveal the spatial orientation of an interacting component to the aromatic plane, the position of the tannin in regard to the aromatic rings of TBZ is most likely on the sides of rings rather than above or below the ring such as in π–π stacking.^35^ In the case of gemin A, this could be caused by the structural hindrance around the dehydrodigalloyl (*m*-GOG) linkage, since the large and upfield Δ*δ* for the H_F_-3 signal could indicate the presence of TBZ parallel to the ring plane of the F galloyl moiety of gemin A (Figure 1), leaving H-1b and H_A_-3 to, and H_G_-3 to be more perpendicularly oriented, coincidently causing a deshielding effect on the protons. Contrary to the mainly upfield Δ*δ*s for aliphatic and aromatic protons, the Δ*δ*s observed for the OHs were systematically downfield, likely due to hydrogen bond formation resulting in a deshielding effect. These effects were observed especially for the OHs of the flavan-3-ols and PC dimers. For the OHs of IVM, these downfield effects were observed as well, confirming the formation of hydrogen bonds between the interacting components.

Although clear interactions between polyphenols and anthelmintics were observed in this study, the levels of the Δ*δ*s remained rather low compared to those typically observed in the interactions between polyphenols and biomacromolecules such as proteins and lipids.^11,36–39,51^ A similar trend was also noted during our previous studies utilizing ITC, where the enthalpy changes observed for the interactions between polyphenols and TBZ were smaller than those previously reported for polyphenol–macromolecule interactions.^33,34,52–56^ As the shift changes observed here were relatively small and could be attributed to noncovalent interactions between the interacting compounds, it would seem unlikely that these weak interactions alone could explain the reduced anthelmintic efficacy in vivo.^28,29^ However, in vivo systems are highly complex, and the chemical environments are influenced by several factors, such as the change of pH and the introduction of various digestive agents along the gastrointestinal tract of an animal. While these factors are taken into account in the development of anthelmintics, they are known to affect the composition and bioavailability of feed polyphenols.^57–59^ The potential impact of these changes in the chemical environment, or the presence of metabolized polyphenols, on the bioavailability and efficacy of commercial anthelmintics remains to be investigated.

## 3 Conclusions

NMR spectroscopy was effective in generating information on the binding sites and strengths and mechanisms of interactions between different polyphenols and two anthelmintics. Hydrogen bonding was the main interaction mechanism for polyphenol interactions with IVM while both hydrogen bond formation and hydrophobic interactions were responsible for interactions between polyphenols and TBZ. The results demonstrated clear differences in the anthelmintic interaction sites of flavan-3-ols, PAs, and HTs; the OH groups in flavan-3-ols and PAs participated greatly in their interactions with anthelmintics, whereas hydrophobic interactions likely also participated in HT interactions with anthelmintics as the Δ*δ*s were more evenly distributed between aromatic and glucose protons. Larger Δ*δ*s, indicating stronger interaction, were observed in the mixtures with TBZ than with IVM in all polyphenol-anthelmintic combinations. The results were also useful in inferring the potential orientations of the polyphenol and the anthelmintic in polyphenol-TBZ interactions via induced ring-current effects. These interactions should be further investigated through in vitro and, eventually, in vivo experiments to better understand their impact on anthelmintic bioavailability within complex biological systems.

## 4 Materials and Methods

### 4.1 Reagents

Analytical grade acetone and methanol were used for the extraction and fractionation of the plant material (≥99%, Sigma-Aldrich, St. Quentin Fallavier, France). HPLC grade methanol (≥99.9%, Sigma-Aldrich, St. Quentin Fallavier, France), acetonitrile (≥99.9%, Honeywell, Seelze, Germany) and formic acid (99–100%, VWR Chemicals, Rosny-sous-Bois, Paris, France) were used for the purification of HTs. LC-MS grade solvents, acetonitrile (OPTIMA, Fisher Scientific, Loughborough, U.K.), formic acid (VWR International, Fontenay-sous-Bois, Paris, France), and absolute ethanol (≥99.8%, VWR International, Fontenay-sous-Bois, Paris, France), were used for ultrahigh-performance liquid chromatography mass spectrometry (UHPLC-MS) analyses. Flavan-3-ols and procyanidin (PC) dimers were purchased from Extrasynthese, Genay, France, with the following purities: (−)-epicatechin (EC), ≥99%; (−)-epigallocatechin (EGC), ≥ 98%; (−)-epicatechin gallate (ECG), ≥97.5%; epigallocatechin gallate (EGCG), ≥98%; PC B2 (epicatechin­(4β→8)­epicatechin), ≥90%, PC A2 (epicatechin­(2β→7;4β→8)­epicatechin), ≥98%. Thiabendazole (PESTANAL, ≥98.0%) and ivermectin (ivermectin B_1a_ ≥ 90%, ivermectin B_1b_ ≤ 5%) were purchased from Sigma-Aldrich International GmbH, St. Louis, MO, USA. Deuterated acetonitrile, CD_3_CN (99.80% D), was purchased from Eurisotop, Saint-Aubin, France. All the water used in the study was type I ultrapure water, produced by a Merck Millipore Synergy UV system (Merck KGaA, Darmstadt, Germany).

### 4.2 Purification of Model Tannins

The purification of model HTs was similar to that previously described.^56,60–63^ The plant materials, presented in Supporting Information (SI) in more detail, were collected into 1 L glass bottles, which were then filled with acetone. The materials were then extracted multiple times with acetone/water (4:1, v/v), and the extraction processes were monitored using an UHPLC instrument coupled to a diode array detector (DAD). After extraction, fractionation of target compounds was conducted utilizing Sephadex LH-20 gel chromatography as previously reported.^56^ Final purification was conducted using preparative and semipreparative high-performance liquid chromatography (HPLC) systems.^56^ The final purity determinations and characterizations of the purified HTs were conducted using UHPLC-DAD-MS/MS and NMR spectroscopy. The instruments used in the analyses, preparation of the samples, and the analytical methods employed are specified in more detail in Sections 4.3 and 4.4.

### 4.3 UHPLC-DAD-MS/MS Analyses

The HTs used have been characterized in our previous study.^33^ Instrumentation for purity determinations consisted of an Acquity UPLC system (Waters Corp., Milford, MA, USA) connected to a Xevo TQ triple-quadrupole mass spectrometer (Waters Corp., Milford, MA, USA). The column used was an Acquity UPLC BEH Phenyl column (2.1 × 100 mm, 1.7 μm, Waters Corp., Wexford, Ireland). The eluents, elution profiles, and electrospray ionization (ESI) parameters for UHPLC-DAD-MS purity determinations were similar to those reported in our previous study.^33^ Sample preparation consisted of diluting a small amount of HT into a suitable amount of 10% aqueous ethanol and filtering the sample through a 0.2 μm polytetrafluoroethylene (PTFE) filter (VWR International, Radnor, PA, USA). The purities of the HTs were determined by integrating peak areas detected at 280 nm and are presented in Table S1.

### 4.4 NMR Analyses

The NMR instrument used was Bruker Avance-III spectrometer operating at 500.12 MHz for ^1^H and at 125.76 MHz for ^13^C. The instrument was equipped with a SmartProbe (Bruker, Fällanden, Switzerland). TopSpin software was used for instrument operation and data analysis (Versions 3.6.5 and 3.5 pl 7,respectively, Bruker, Billerica, MA, USA). Because of the limited solubility of the anthelmintics, *d*_3_-acetonitrile was used as the solvent. The residual solvent signals were used as chemical shift references, *δ*_H_ = 1.94 ppm and *δ*_C_ = 118.69 ppm.

For determining the Δ*δ*s of the proton signals in the polyphenol-anthelmintic mixtures, first the signals of the pure compounds (polyphenols and commercial anthelmintics) were assigned by measuring their one-dimensional ^1^H NMR and ^13^C NMR spectra and various 2D correlation spectra. Homonuclear correlation spectroscopy (COSY), rotating-frame nuclear Overhauser effect correlation spectroscopy (ROESY, with 200 ms mixing time), multiplicity-edited heteronuclear single quantum coherence (HSQC), heteronuclear multiple bond correlation (HMBC), and selective HMBC (carbonyl region, 166 ± 5 ppm) experiments were performed to aid the signal assignments. Because of the hindered rotation of the interflavanoid bond in PC B2 that broadens the signals at 298 K, the dimer was characterized at 243 K where the conformational equilibrium reaction is slowed down and sharp spectra for two separate conformations are observed.^46,49^ Additional spectra, i.e., ROESY and HMBC, were measured for mixtures of polyphenols and anthelmintics if needed, e.g., for the characterization of separated OH signals observed for the flavan-3-ols and PC A2.

The assignment of the NMR signals of the studied polyphenols in *d*_3_-acetonitrile followed the examples from previous publications.^17,37,46,47,64,65^ For HTs, the assignment began from the glucose protons after which the obtained information was used to determine the signals for the adjacent galloyl, HHDP, and NHTP groups by utilizing the information collected via HMBC and ROESY spectra.^17^ Similar approach was used with flavan-3-ols and PAs, with the signal assignment starting from the C ring protons of the structures. ^1^H NMR signal assignments for all model polyphenols and commercial anthelmintics are presented in Supporting Information (Figures S1–S13).

The interaction studies were performed by recording the ^1^H NMR spectra of mixtures containing different molar ratios of each of the two commercial anthelmintics to each polyphenol, with the concentration of the polyphenol always kept constant at 0.3 mM. The polyphenol to anthelmintic molar ratios in the interaction studies of IVM were 3:1, 1:1, 1:3, 1:5, and 1:10, and the corresponding molar ratios in the studies of TBZ were 3:1, 1:1, 1:3, and 1:5. Chemical shift changes for the compounds in the mixtures with respect to the corresponding chemical shifts of the pure compounds were calculated by using the equation Δ*δ* = *δ*_mixture_ – *δ*_pure compound_ and plotted as a function of molar ratio. The interaction studies were performed at 298 K, except for PC B2, which were performed at both 298 and 243 K.

## Supplementary Material



## Notes

Information on the plant origin and purity of the HTs, as well as the ^1^H NMR spectra of the pure compounds; Δ*δ*s are also shown for each polyphenol-anthelmintic mixture at different molar ratios (PDF)

## Nomenclature

COSY: homonuclear correlation spectroscopy
CSP: chemical shift perturbance
DAD: diode array detector
EC: epicatechin
ECG: epicatechin gallate
EGC: epigallocatechin
EGCG: epigallocatechin gallate
ESI: electrospray ionization
GOG: dehydrodigalloyl
HHDP: hexahydroxydiphenoyl
HMBC: heteronuclear multiple bond correlation
HPLC: high-performance liquid chromatography
HSQC: heteronuclear single quantum coherence
HT: hydrolyzable tannin
ITC: isothermal titration calorimetry
IVM: ivermectin
LC: liquid chromatography
MS: mass spectrometry
NMR: nuclear magnetic resonance
PA: proanthocyanidin
PC: procyanidin
PGG: pentagalloylglucose
PTFE: polytetrafluoroethylene
ROESY: rotating-frame nuclear Overhouser effect correlation spectroscopy
TBZ: thiabendazole
UHPLC: ultrahigh-performance liquid chromatography
