# Supplementary material for: Molecular Aspects of Tannin-Anthelmintic Interactions as Revealed by NMR Spectroscopy
Source: ACS Omega. 2025 Jul 15;10(29):32174–88. doi: 10.1021/acsomega.5c03937 (PMC12311743; doi:10.1021/acsomega.5c03937)
Supplement: Supplementary file 1 [file ao5c03937_si_001.pdf]

Molecular Aspects of Tannin-Anthelmintic Interactions as Revealed by NMR Spectroscopy

Mimosa Sillanpää*, Petri Tähtinen, and Maarit Karonen

Department of Chemistry, University of Turku, FI-20014 Turku, Finland

*Email: mamsil@utu.fi

Table of Contents

Table S1. 3
Figure S1..4
Figure S2..5
Figure S3..6
Figure S4..7
Figure S5..8
Figure S6..9
Figure S7..10
Figure S8..11
Figure S9..12
Figure S10..13
Figure S11..15
Figure S12..14
Figure S13..16
Figure S14..17
Figure S15..18
Figure S16..19
Figure S17..20
Figure S18..21
Figure S19..22

Figure S20. 23
Figure S21 24
Figure S22. 25
Figure S23. 27
Figure S24. 26
Figure S25. 28

Table S1. Plant origin and purity and calculated and measured molecular weights of the hydrolysable tannins.

Plant material			Target compound				
**Common name**	**Scientific name**	**Plant part**	**Tannin**	**Purity****	**Mcalculated**	**Mmeasured**	**Error (ppm)**
Meadowsweet	*Filipendula ulmaria*	inflorescence	tellimagrandin I	96 %	786.09060	786.09027	-0.41725
Purple loosestrife	*Lythrum salicaria*	inflorescence and leaves	vescalagin	84 %	934.07020	934.06846	-1.86067
Meadowsweet	*Filipendula ulmaria*	inflorescence	tellimagrandin II	99 %	938.10140	938.09987	-1.62882
*	-	-	pentagalloylglucose	99 %	940.11700	940.11454	-2.61457
Herb bennet	*Geum urbanum*	leaves	gemin A	97 %	1872.17160	1872.16876	-1.51482

*Pentagalloylglucose was prepared via methanolysis from commercial gallic acid purchased from J.T. Baker (Denver, Netherlands).
**Determined by UV at 280 nm.

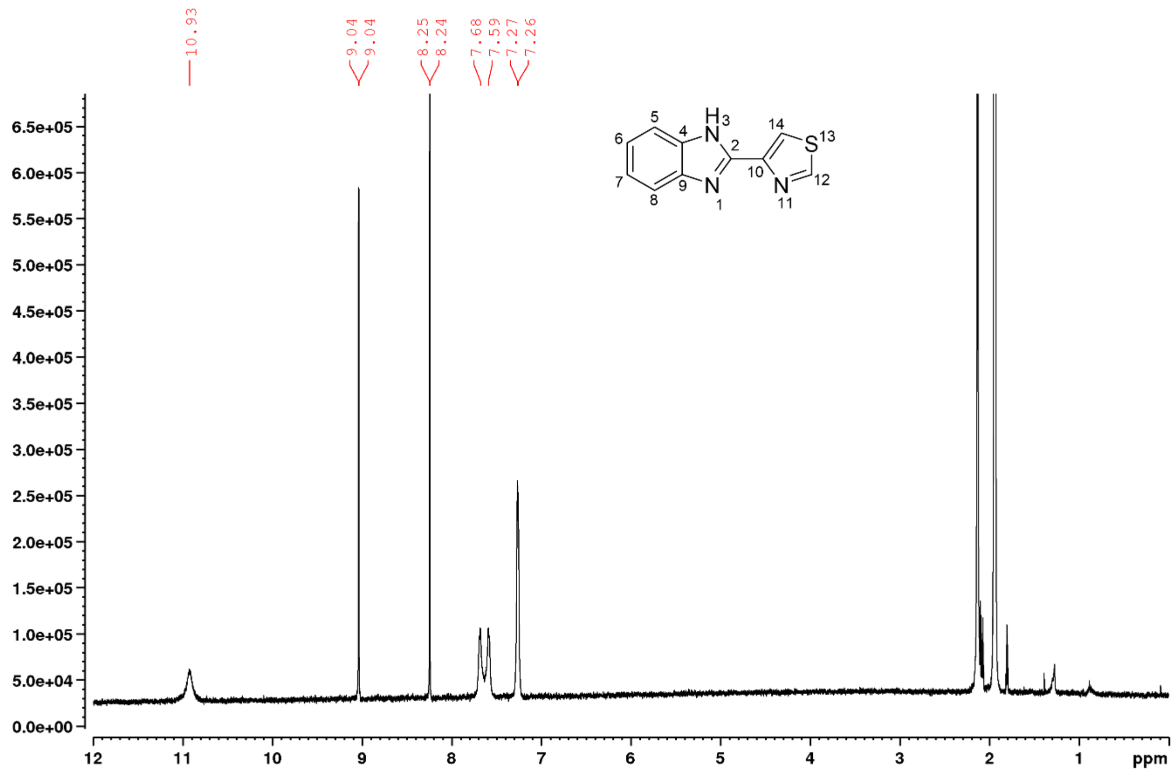

Figure S1. ^{1}H NMR (500 MHz, CD_3CN) of thiabendazole: δ 10.93 (s, 1, H-3), 9.04 (d, 1, J = 2.0 Hz, H-12), 8.24 (d, 1, J = 2.0 Hz, H-14), 7.68 (br, 1, H-5/H-8), 7.59 (br, 1, H-5/H-8), 7.27 (br, 1, H-6/H-7), 7.26 (br, 1, H-6/H-7).

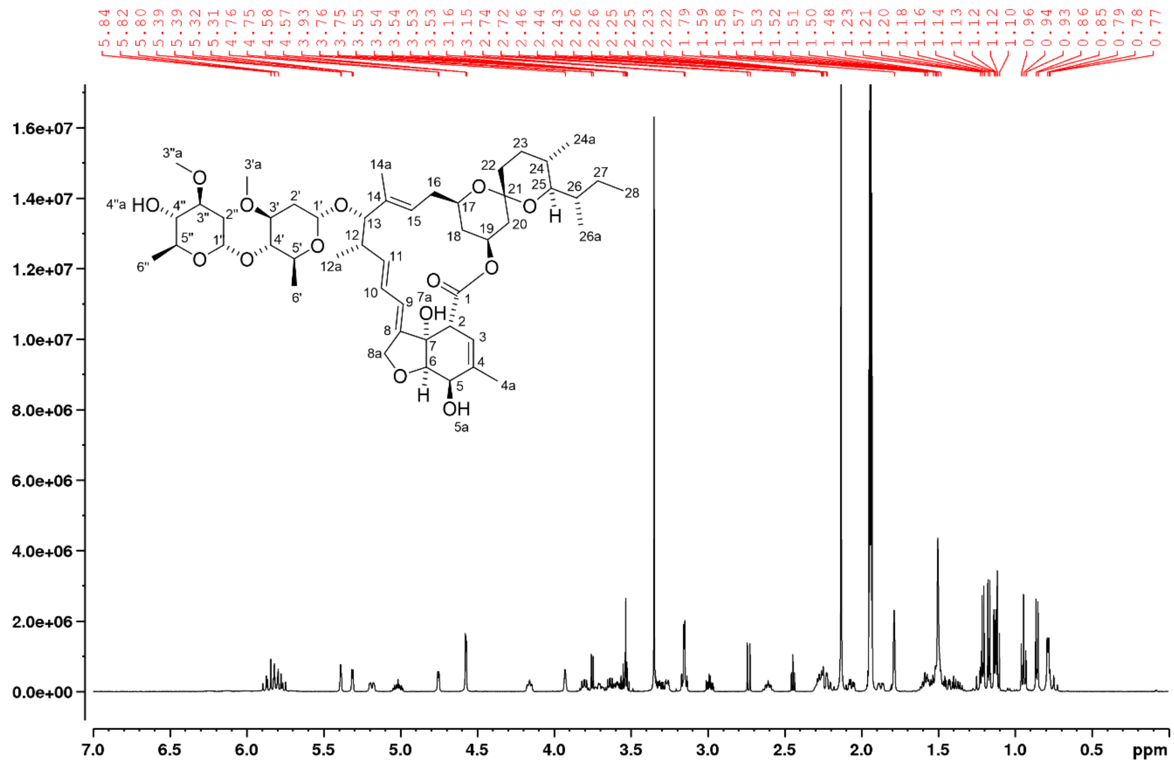

Figure S2. ^{1}H NMR (500 MHz, CD_3CN) of ivermectin: δ 5.82 (m, 3, J = 6.7 Hz, H-10, H-9, H-11), 5.39 (d, 1, J = 1.5 Hz, H-3), 5.31 (d, 1, J = 3.1 Hz, H-1''), 5.19 (q, 1, J = 3.6 Hz, H-15), 5.02 (m, 1, J = 5.4 Hz, H-19), 4.75 (d, 1, J = 2.7 Hz, H-1'), 4.57 (d, 2, J = 2.1 Hz, H-8a), 4.16 (t, 1, J = 7.3 Hz, H-5), 3.93 (s, 1, H-13), 3.80 (q, 1, J = 5.2 Hz, H-5'), 3.75 (d, 1, J = 5.7 Hz, H-6), 3.71 (m, 1, J = 3.3 Hz, H-17), 3.64 (q, 1, J = 5.2 Hz, H-5''), 3.59 (m, 1, J = 2.8 Hz, H-3'), 3.54 (dq, 1.2, J = 7.0, 5.3 Hz, $CH_3\underline{CH_2}OH$ impurity), 3.53 (s, 1, OH-7a), 3.35 (s, 6, H-3'a, 3''a), 3.31 (m, 1, J = 3.3 Hz, H-3''), 3.26 (d, 1, J = 7.1 Hz, H-25), 3.15 (d, 1, J = 4.0 Hz, OH-4''a), 3.15 (dd, 1, J = 10.9, 7.0 Hz, H-4'), 3.15 (m, 1, H-2), 2.98 (dt, 1, J = 4.1, 13.6 Hz, H-4''), 2.73 (d, 1, J = 8.8 Hz, OH-5a), 2.61 (m, 1, J = 3.2 Hz, H-12), 2.44 (t, 0.6, J = 5.28 Hz, $CH_3CH_2\underline{OH}$ impurity), 2.23 (m, 4, J = 5.0 Hz, H-2'$_{eq}$, H-16, H-2''$_{eq}$), 2.06 (m, 1, J = 2.3 Hz, H-20$_{eq}$), 1.87 (m, 1, J = 3.5 Hz, H-18$_{eq}$), 1.79 (s, 3, H-4a), 1.57 (m, 3, J = 4.3 Hz, H-26, H-23), 1.50 (s, 6, H-14a, H-22, H-24), 1.41 (m, 3, J = 4.9 Hz, H-2'$_{ax}$, H-2''$_{ax}$, H-27), 1.23 (t, 1, J = 11.9 Hz, H-20$_{ax}$), 1.21 (d, 3, J = 6.2 Hz, H-6'), 1.17 (d, 3, J = 6.3 Hz, H-6''), 1.13 (d, 3, J = 7.0 Hz, H-12a), 1.12 (t, 2, J = 7.0 Hz, $\underline{CH_3}CH_2OH$ impurity), 0.94 (t, 3, J = 7.3 Hz, H-28), 0.86 (d, 3, J = 6.7 Hz, H-26a), 0.79 (d, 3, J = 5.4 Hz, H-24a), 0.75 (t, 1, J = 12.2 Hz, H-18$_{ax}$).

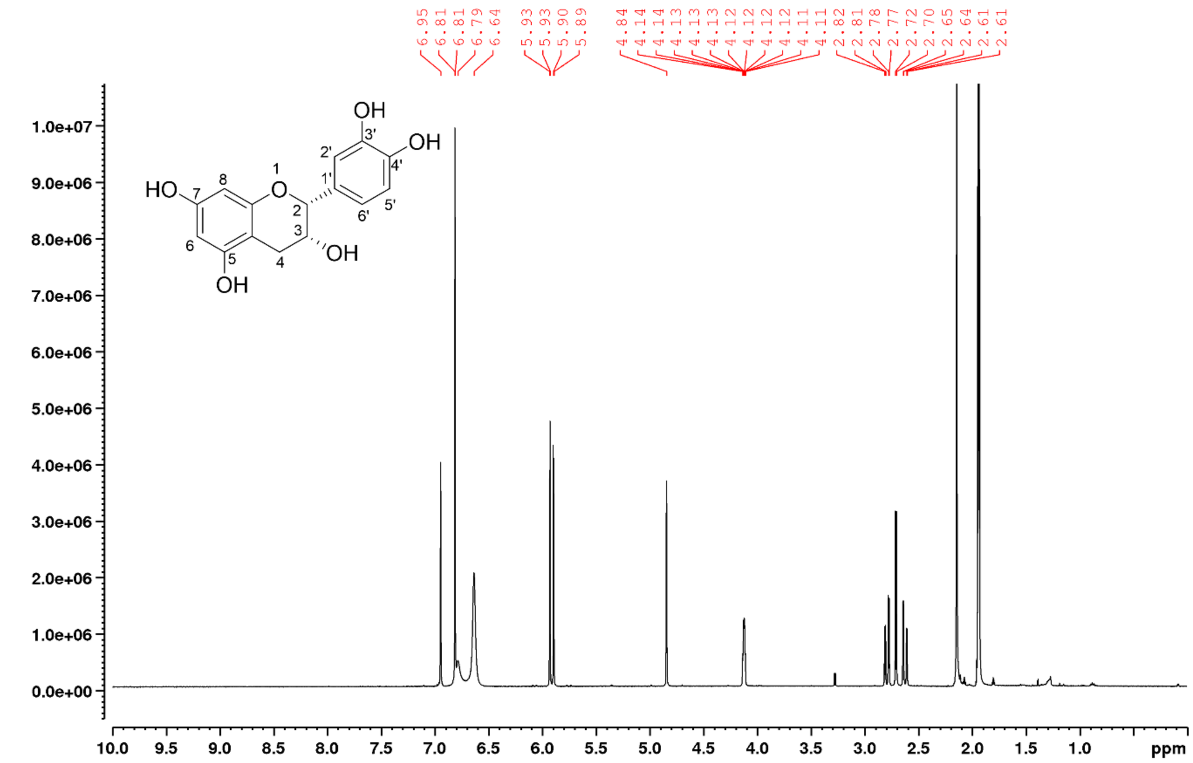

Figure S3. ^{1}H NMR (500 MHz, CD_3CN) of epicatechin: δ 6.95 (s, 1, H-2'), 6.81 (d, 2, J = 1.1 Hz, H-5'/H-6'), 6.79 (br, 1, OH-5), 6.64 (br, 3, OH-3', OH-4', OH-7), 5.93 (d, 1, J = 2.4 Hz, H-6), 5.90 (d, 1, J = 2.4 Hz, H-8), 4.84 (s/br, 1, H-2), 4.12 (m, 1, J = 1.4 Hz, H-3), 2.79 (dd, 1, J = 4.5, 16.7 Hz, H-4β), 2.71 (d, 1, J = 5.6 Hz, OH-3), 2.63 (dd, 1, J = 2.6, 16.8 Hz, H-4α).

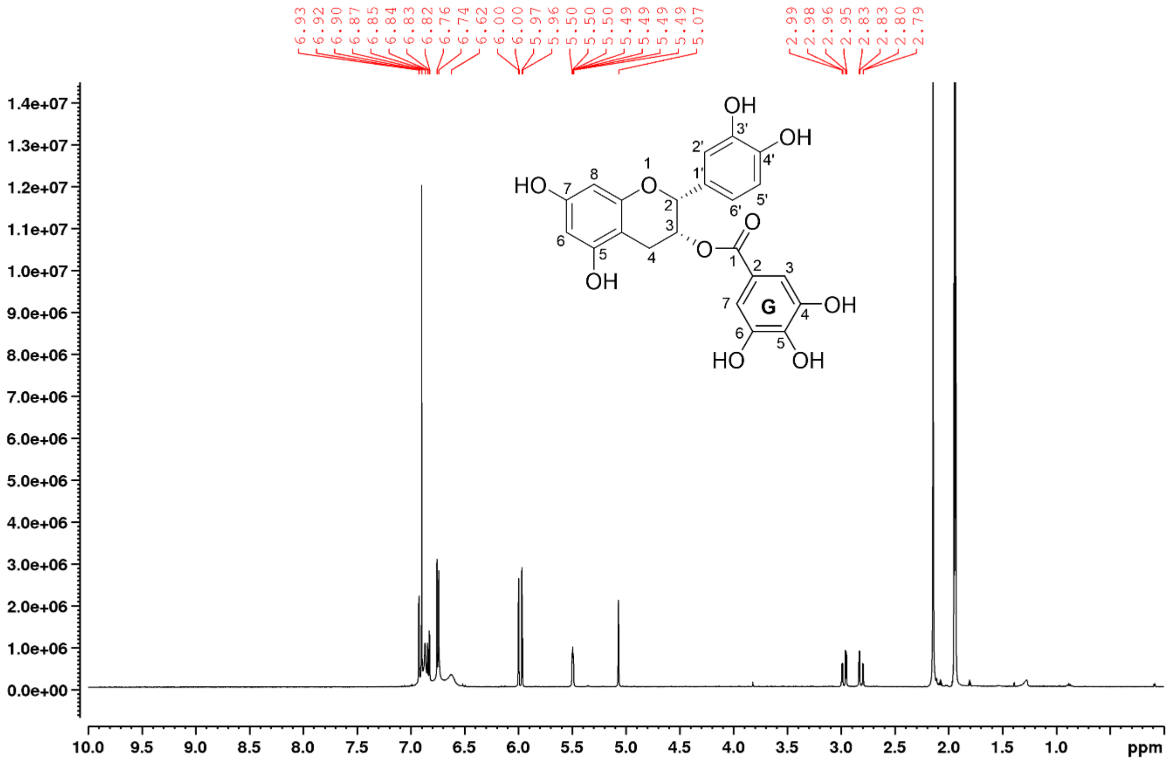

Figure S4. ^{1}H NMR (500 MHz, CD_3CN) of epicatechin gallate: *δ* 6.92 (d, 1, *J* = 2.0 Hz, H-2’), 6.90 (s, 2, H_G-3, H_G-7), 6.87 (br, 3, OH-5, OH_G-4, OH_G-6), 6.83 (dd, 1, *J* = 1.7, 8.2 Hz, H-6’), 6.75 (d, 1, J=8.2 Hz, H-5’), 6.74 (br, 2, OH-7, OH_G-5), 6.62 (br, 2, OH-3’, OH-4’), 6.00 (d, 1, *J* = 2.3 Hz, H-8), 5.97 (d, 1, *J* = 2.3 Hz, H-6), 5.49 (m, 1, *J* = 1.3 Hz, H-3), 5.07 (s/br, 1, H-2), 2.97 (dd, 1, *J* = 4.5, 17.4 Hz, H-4β), 2.81 (dd, 1, *J* = 2.1, 17.5 Hz, H-4α).

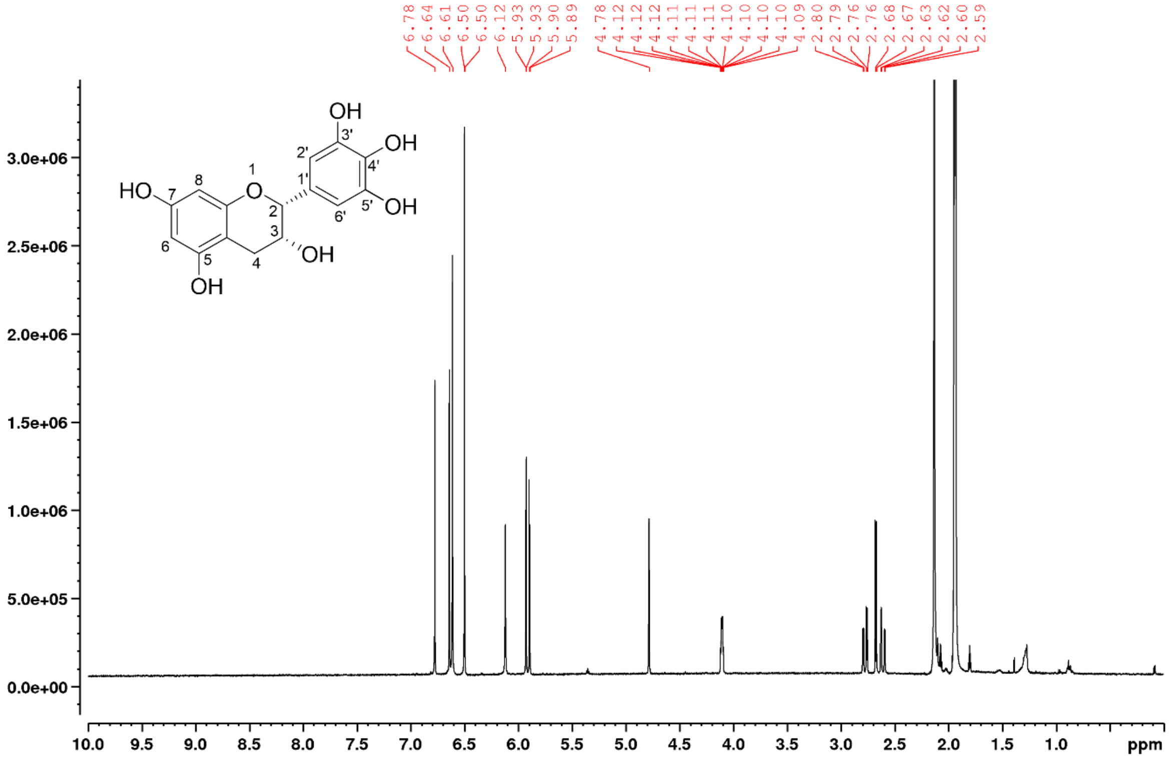

Figure S5. ^{1}H NMR (500 MHz, CD_3CN) of epigallocatechin: δ 6.78 (s, 1, OH-5), 6.64 (s, 1, OH-7), 6.61 (s, 2, OH-3’, OH-5’), 6.50 (d, 2, J = 0.4 Hz, H-2’, H-5’), 6.12 (s, 1, OH-4’), 5.93 (d, 1, J = 2.3 Hz, H-6), 5.90 (d, 1, J = 2.3 Hz, H-8), 4.78 (br, 1, H-2), 4.11 (m, 1, J = 1.4 Hz, H-3), 2.78 (dd, 1, J = 4.5, 16.7 Hz, H-4β), 2.68 (d, 1, J = 5.4 Hz, OH-3), 2.61 (dd, 1, J = 2.7, 16.7 Hz, H-4α).

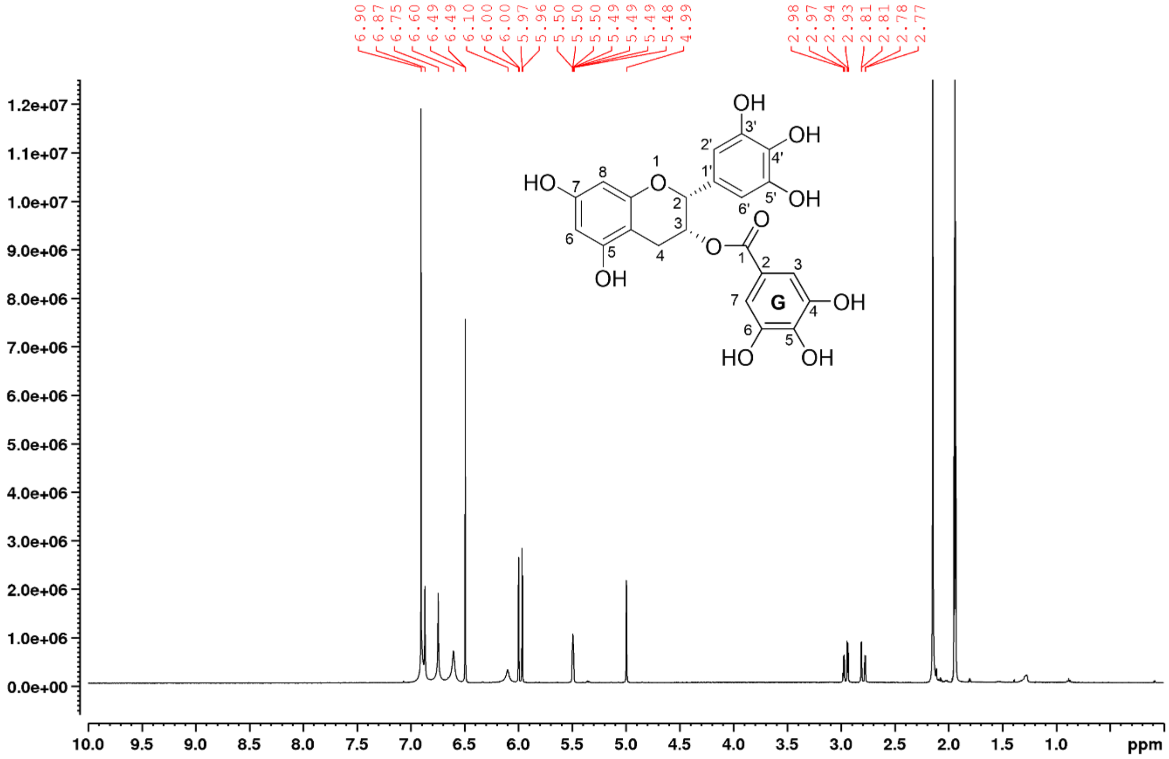

Figure S6. ^{1}H NMR (500 MHz, CD_3CN) of epigallocatechin gallate: δ 6.90 (s/br, 4, HG-3, HG-7, OHG-4, OHG-6), 6.87 (s, 1, OH-5), 6.75 (br, 2, OH-7, OHG-5), 6.60 (br, 2, OH-3', OH-5'), 6.49 (d, 2, J = 0.3 Hz, H-2', H-6'), 6.10 (br, 1, OH-4'), 6.00 (d, 1, J = 2.3 Hz, H-8), 5.96 (d, 1, J = 2.3 Hz, H-6), 5.49 (m, 1, J = 1.3 Hz, H-3), 4.99 (s/br, 1, H-2), 2.96 (dd, 1, J = 4.5, 17.3 Hz, H-4β), 2.79 (dd, 1, J = 2.2, 17.5 Hz, H-4α).

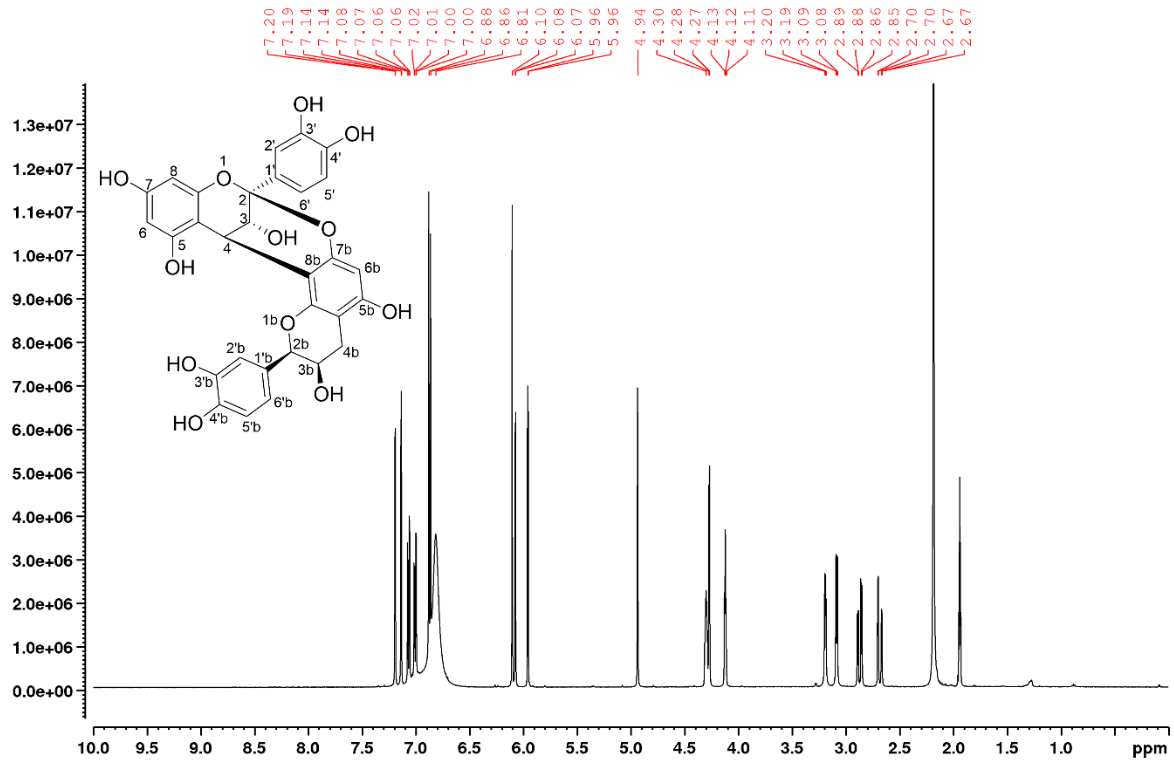

Figure S7. ^{1}H NMR (500 MHz, CD_3CN) of procyanidin A2: δ 7.19 (d, 1, J = 2.0 Hz, H-2'b), 7.14 (d, 1, J = 2.1 Hz, H-2'), 7.07 (dd, 1, J = 2.2, 8.3 Hz, H-6'), 7.01 (dd, 1, J = 2.0, 8.2 Hz, H-6'b), 6.87 (d, 2, J=8.3 Hz, H-5', H-5'b), 6.81 (br, 7, OH-3', OH-3'b, OH-4', OH-4'b, OH-5, OH-5b, OH-7), 6.10 (s, 1, H-6b), 6.08 (d, 1, J = 2.3 Hz, H-6), 5.96 (d, 1, J = 2.4 Hz, H-8), 4.94 (s, 1, H-2b), 4.30 (br, 1, H-3b), 4.27 (d, 1, J = 3.5 Hz, H-4), 4.12 (t, 1, J = 3.8 Hz, H-3), 3.19 (d, 1, J = 4.6 Hz, OH-3), 3.09 (d, 1, J = 6.0 Hz, OH-3b), 2.87 (dd, 1, J = 4.6, 17.2 Hz, H-4bβ), 2.68 (dd, 1, J = 1.5, 17.3 Hz, H-4bα).

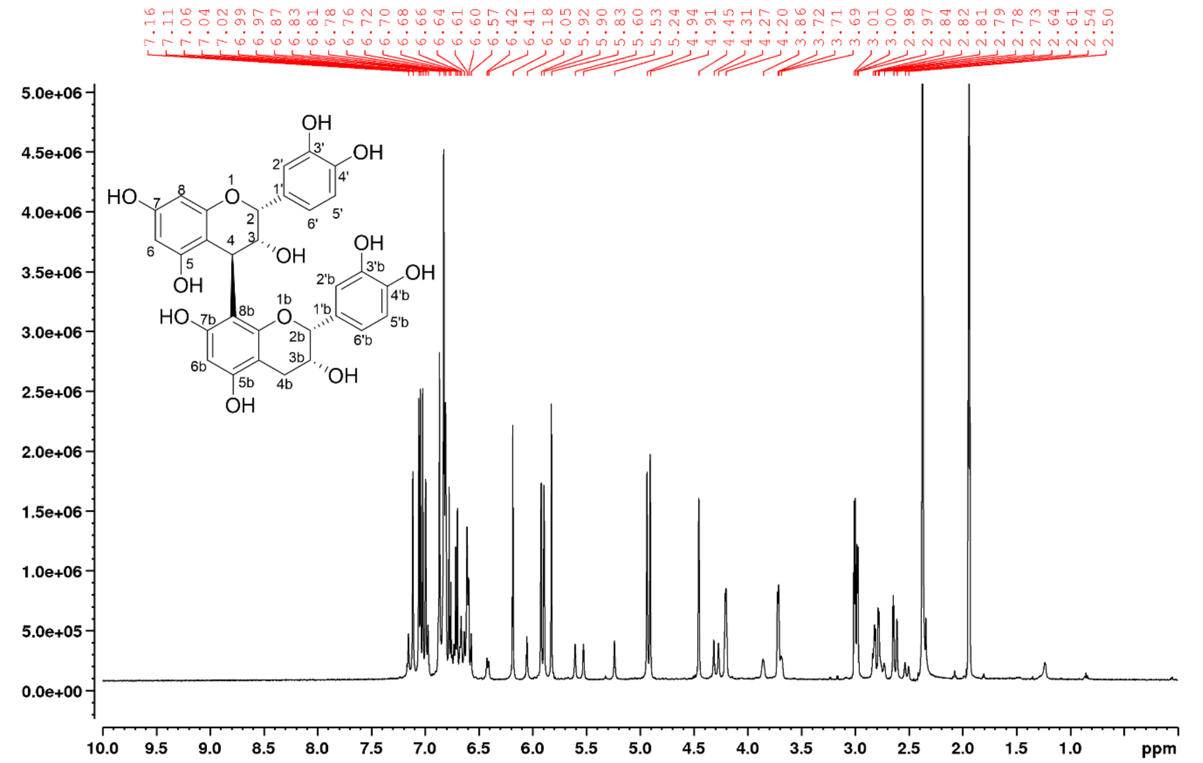

Figure S8. ^{1}H NMR (500 MHz, CD_3CN, 243 K) of procyanidin B2: δ 7.11 (s, 1, OH-5), 7.06 (s, 1, OH-3'/OH-4'), 7.04 (s, 1, OH-7), 7.02 (s, 1, OH-5b), 6.99 (s, 1, H-2'b/H-6'b/OH-3'/OH-4'), 6.87 (s, 1, OH-3'b/OH-4'b), 6.83 (s, 2, OH-3'b/OH-4'b/H-2'/H-2'b), 6.81 (br, 2, H-2'/H-2'b/OH-3'b/OH-4'b), 6.77 (d, J = 8.2 Hz, 1,H-5'/H-5'b), 6.71 (d, J = 8.2 Hz, 1, H-5'/H-5'b), 6.60 (d, J = 7.3 Hz, 1/2, H-6'/H-6'b), 6.18 (s, 1, OH-7b), 5.92 (s, 1, H-8), 5.90 (s, 1, H-6), 5.83 (s, 1, H-6b), 4.94 (s, 1, H-2b), 4.91 (s, 1, H-2), 4.45 (s, 1, H-4), 4.20 (s, 1, H-3b), 3.72 (d, 1, J = 4.8 Hz, H-3), 3.00 (d, 1, J = 5.5 Hz, OH-3), 2.98 (d, 1, J = 4.2 Hz, OH-3b), 2.80 (dd, 1, J = 4.4, 17.1 Hz, H-4bβ), 2.63 (d, 1, J = 16.3 Hz, H-4bα). Signal assignments are presented for the major rotamer.

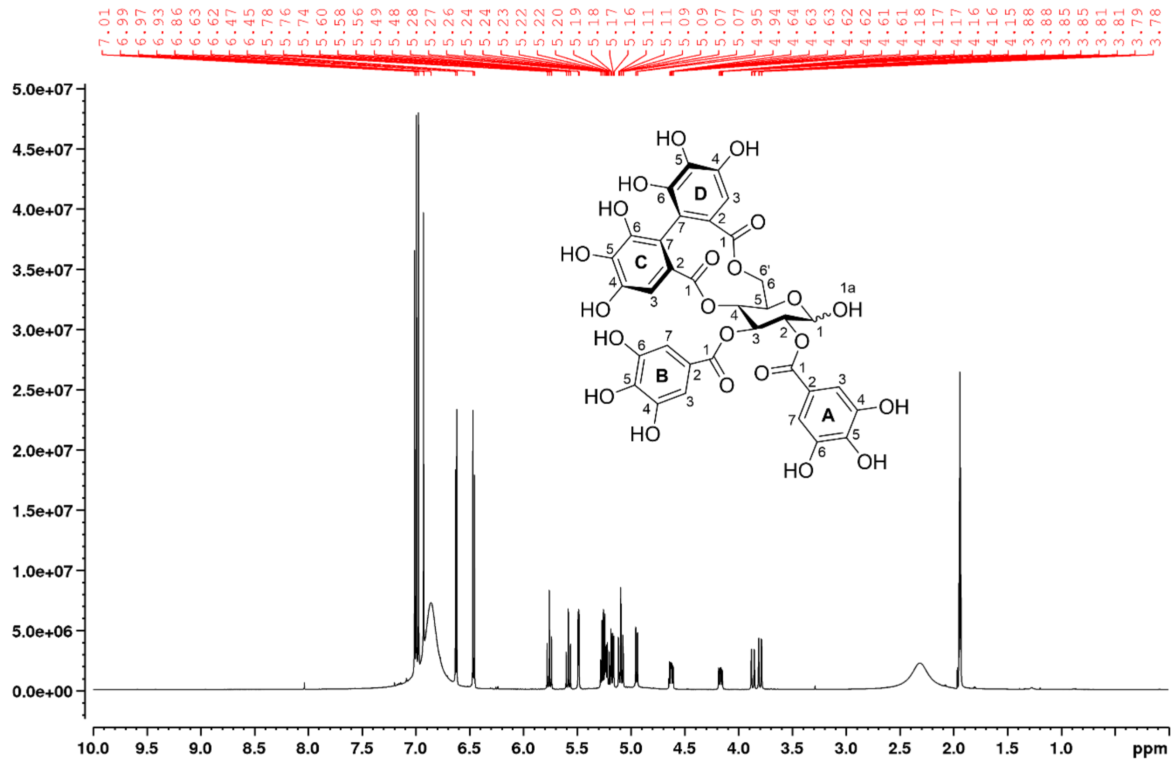

Figure S9. ^{1}H NMR (500 MHz, CD_3CN) of tellimagrandin I: δ 7.01 (s, 2, H_A-3β, H_A-7β), 6.99 (s, 2, H_A-3α, H_A-7α), 6.97 (s, H_B-3α, H_B-7α), 6.93 (s, 2, H_B-3β, H_B-7β), 6.86 (br, 24, OH), 6.63 (s, 1, H_D-3β), 6.62 (s, 1, H_D-3α), 6.47 (s, 1, H_C-3α), 6.45 (s, 1, H_C-3β), 5.76 (t, J = 10.0 Hz, 1, H-3α), 5.58 (t, 1, J = 9.7 Hz, H-3β), 5.49 (d, 1, J = 3.8 Hz, H-1α), 5.27 (t, 1, J = 6.6 Hz, H-6β), 5.24 (t, 1, J = 6.5 Hz, H-6α), 5.22 (dd, 1, J = 9.6, 8.0 Hz, H-2β), 5.17 (dd, 1, J = 3.8, 10.0 Hz, H-2α), 5.09 (t, 1, J = 10.1 Hz, H-4β), 5.09 (t, 1, J = 9.9 Hz, H-4α), 4.95 (d, 1, J = 8.0 Hz, H-1β), 4.62 (ddd, 1, J = 1.1, 6.7, 10.2 Hz, H-5α), 4.17 (ddd, 1, J = 1.1, 6.6, 10.0 Hz, H-5β), 3.87 (dd, 1, J = 1.1, 13.2 Hz, H-6’β), 3.80 (dd, 1, J = 1.4, 13.2 Hz, H-6’α).

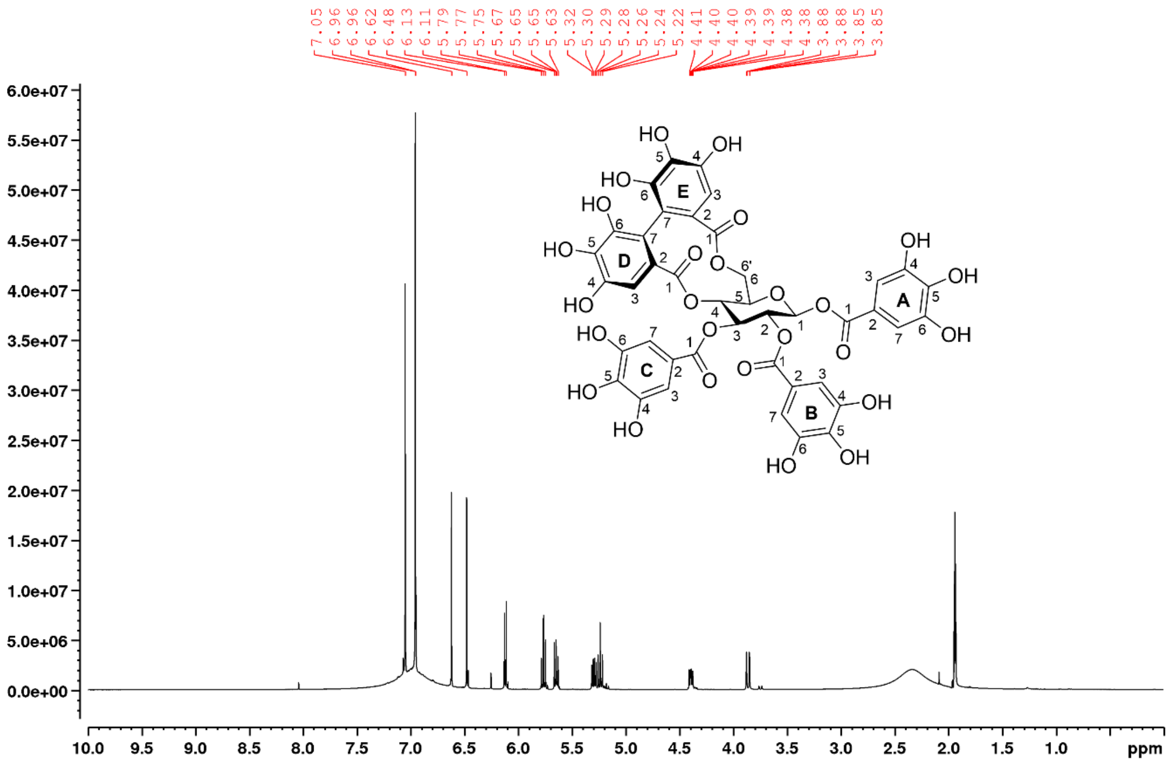

Figure S10. ^{1}H NMR (500 MHz, CD_3CN) of tellimagrandin II: δ 7.05 (s, 2, H_A-3, H_A-7), 7.00 (br, 15, OH), 6.96 (s, 4, H_B-3, H_B-7, H_C-3, H_C-7), 6.62 (s, 1, H_E-3), 6.48 (s, 1, H_D-3), 6.12 (d, 1, *J* = 8.2 Hz, H-1), 5.77 (t, 1, *J* = 9.6 Hz, H-3), 5.65 (dd, 1, *J* = 5.9 Hz, H-2), 5.30 (dd, 1, *J* = 6.7 Hz, H-6), 5.24 (t, 1, *J* = 9.9 Hz, H-4), 4.40 (dd, 1, *J* = 1.1, 6.6, 10.1 Hz, H-5), 3.87 (d, 1, *J* = 4.9 Hz, H-6').

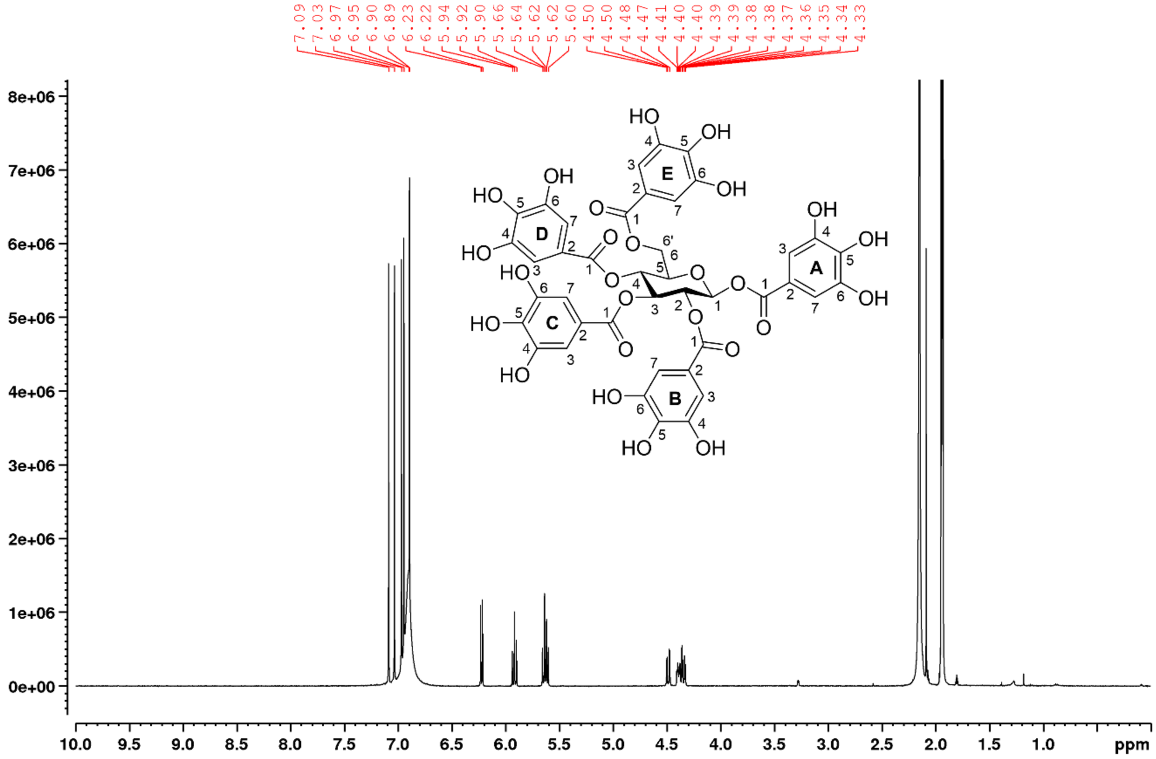

Figure S11. ^{1}H NMR (500 MHz, CD_3CN) of pentagalloylglucose: δ 7.09 (s, 2, H_E-3, H_E-7), 7.03 (s, 2, H_A-3, H_A-7), 6.97 (s, 2, H_D-3, H_D-7), 6.95 (s, 2, H_B-3, H_B-7), 6.90 (br, 15, OH), 6.89 (s, 2, H_C-3, H_C-7), 6.22 (d, 1, J = 8.3 Hz, H-1), 5.92 (t, 1, J = 9.7 Hz, H-3), 5.64 (t, 1, J = 9.5 Hz, H-4), 5.61 (d, 1, J = 9.8 Hz, H-2), 4.49 (dd, 1, J = 2.1, 12.3 Hz, H-6), 4.39 (m, 1, J = 2.4 Hz, H-5), 4.34 (dd, 1, J = 4.5, 12.3 Hz, H-6').

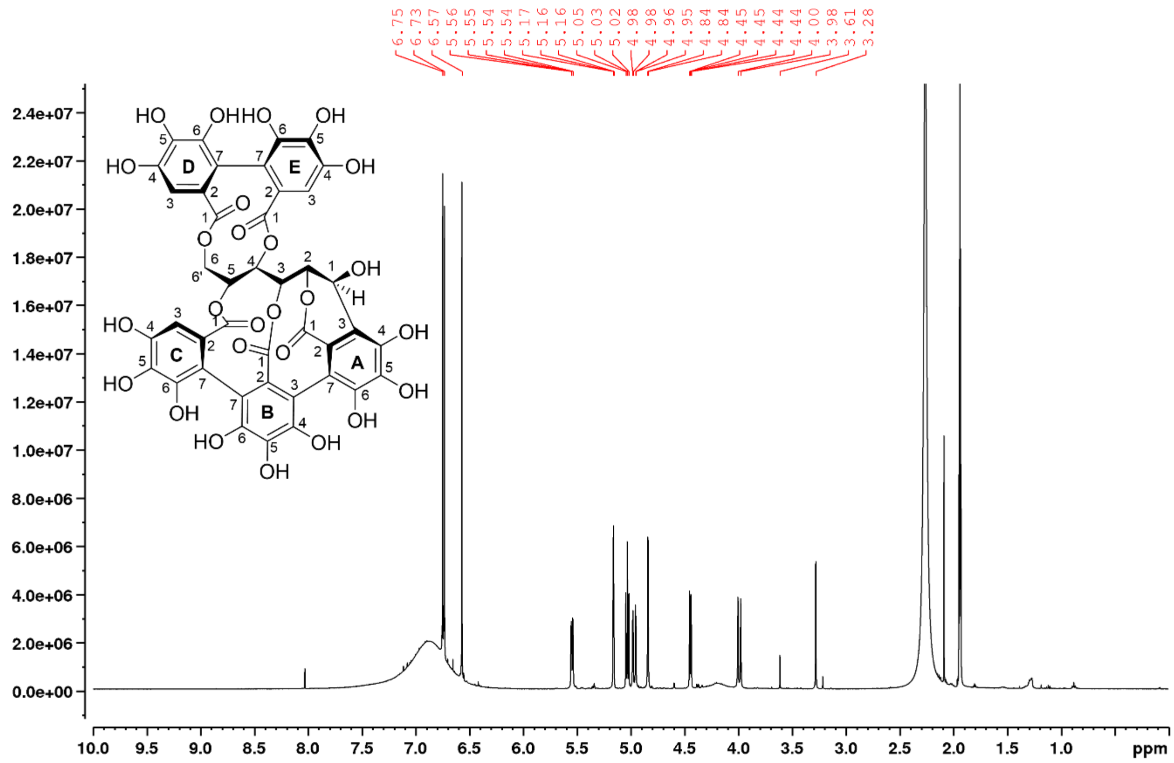

Figure S12. ^{1}H NMR (500 MHz, CD_3CN) of vescalagin: δ 6.88 (br, 16, OH), 6.75 (s, 1, H_C-7), 6.73 (s, 1, H_E-3), 6.57 (s, 1, H_D-3), 5.55 (dd, 1, J = 1.0, 7.3 Hz, H-5), 5.16 (t, 1, J = 1.8 Hz, H-2), 5.03 (t, 1, J = 7.0 Hz, H-4), 4.97 (dd, 1, J = 2.5, 13.1 Hz, H-6), 4.84 (d, 1, J = 2.1 Hz, H-1), 4.45 (dd, 1, J = 1.4, 6.7 Hz, H-3), 3.99 (d, 1, J = 12.4 Hz, H-6’).

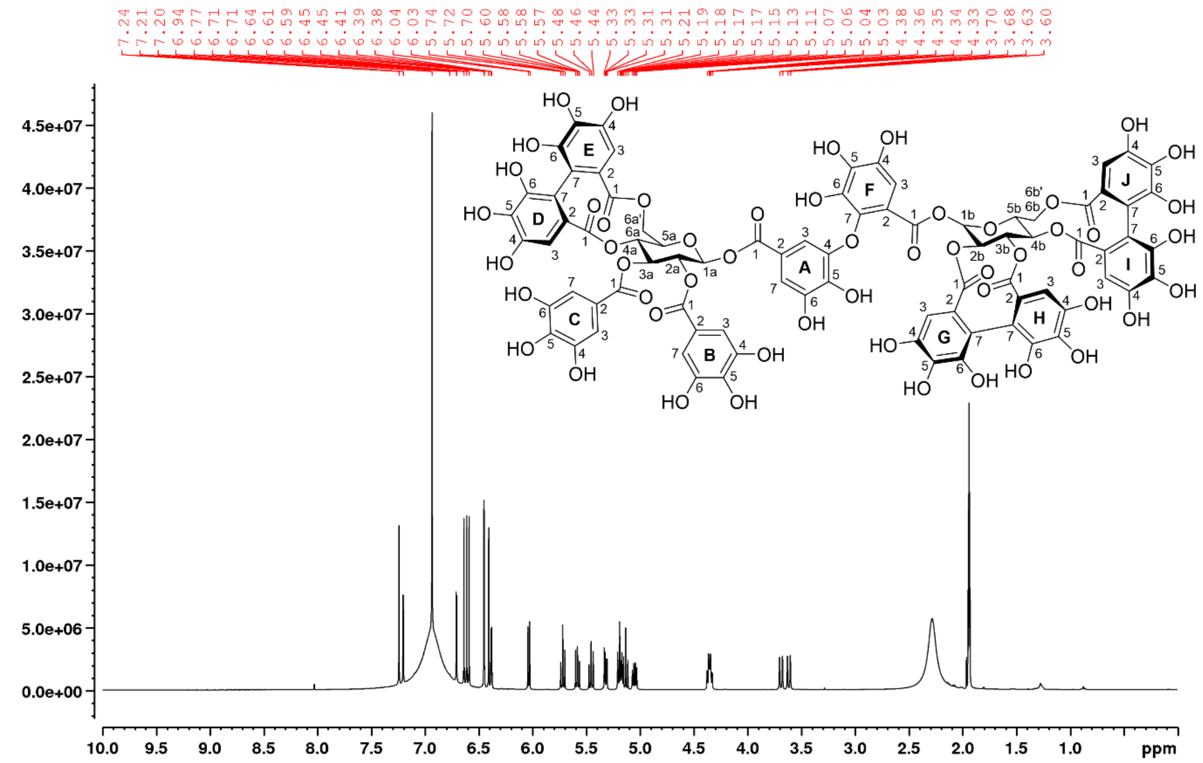

Figure S13. ^{1}H NMR (500 MHz, CD_3CN) of gemin A: δ 7.24 (s, 1, H_F-3), 7.20 (d, 1, J = 1.9 Hz, H_A-7), 6.94 (s, 4, H_B-3, H_B-7, H_C-3, H_B-7), 6.94 (br, 29, OH), 6.71 (d, 1, J = 2.0 Hz, H_A-3), 6.64 (s, 1, H_I-3), 6.61 (s, 1, H_J-3), 6.59 (s, 1, H_E-3), 6.45 (s, 1, H_H-3), 6.45 (s, 1, H_D-3), 6.41 (s, 1, H_G-3), 6.38 (d, 1, J = 4.0 Hz, H-1b), 6.04 (d, 1, J = 8.2 Hz, H-1a), 5.72 (t, 1, J = 9.6 Hz, H-3a), 5.58 (dd, 1, J = 9.5, 8.3 Hz, H-2a), 5.46 (t, 1, J = 9.9 Hz, H-3b), 5.32 (dd, 1, J=4.05, 9.40 Hz, H-2b), 5.19 (t, 1, J = 9.9 Hz, H-4a), 5.19 (q, 1, J = 6.7 Hz, H-6a), 5.13 (t, 1, J = 10.3 Hz, H-4b), 5.05 (q, 1, J = 6.7 Hz, H-6b), 4.35 (m, 2, J = 5.8 Hz, H-5b, H-5a), 3.69 (d, 2, J = 12.7 Hz, H-6'a), 3.62 (d, 1, J = 12.6 Hz, H-6'b).

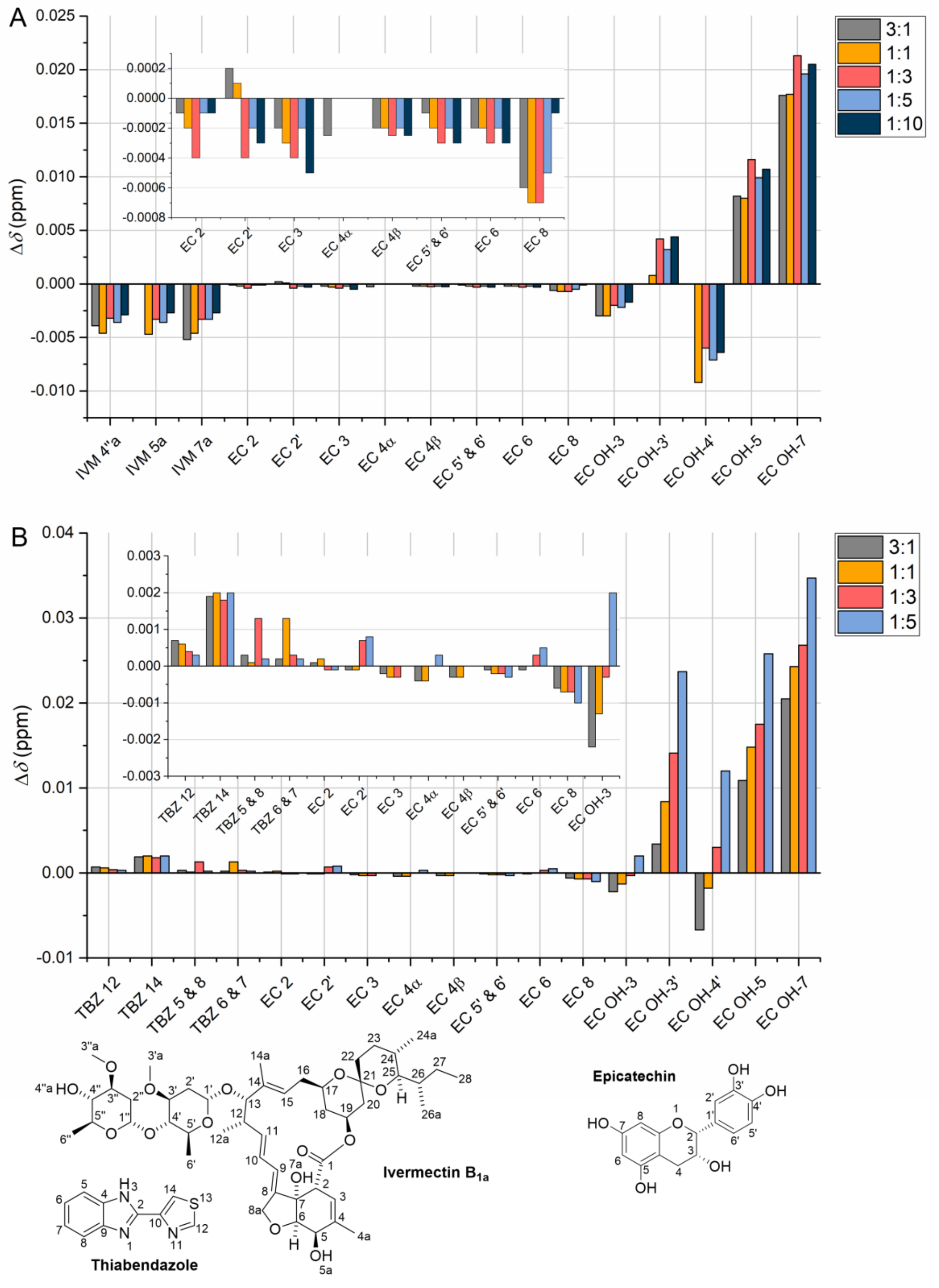

Figure S14. Changes in the chemical shifts ($\Delta\delta$, $\Delta\delta = \delta_{\text{mixture}} - \delta_{\text{pure compound}}$) due to the interactions between epicatechin (EC) and ivermectin (IVM, A) and thiabendazole (TBZ, B) at different molar ratios of the polyphenol to the anthelmintic. $\Delta\delta$s are shown for selected signals of the anthelmintic and for all signals of the polyphenol. OH-3’ and OH-4’ of EC could not be separated at the lowest molar ratio of IVM (3:1) and thus $\Delta\delta$s for these signals could not be calculated. Other missing data are due to overlapping signals of the components or because $\Delta\delta$ was zero.

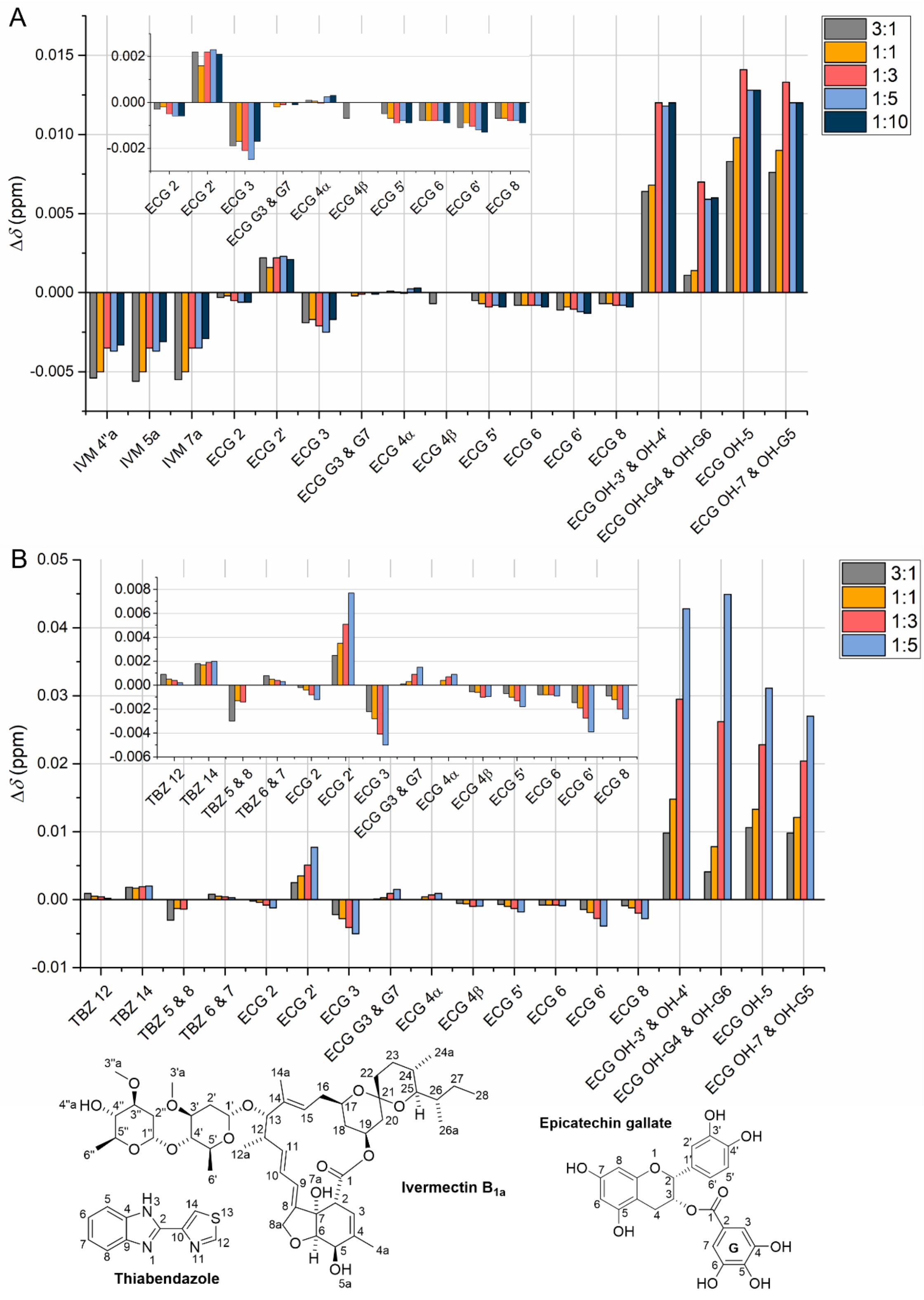

Figure S15. Changes in the chemical shifts (Δ*δ*s, $\Delta\delta = \delta_{mixture} - \delta_{pure\ compound}$) due to the interactions between epicatechin gallate (ECG) and ivermectin (IVM, A) and thiabendazole (TBZ, B) at different molar ratios of the polyphenol to the anthelmintic. Δ*δ*s are shown for selected signals of the anthelmintic and for all signals of the polyphenol. Missing data are due to overlapping signals of the components or because no chemical shift changes were observed.

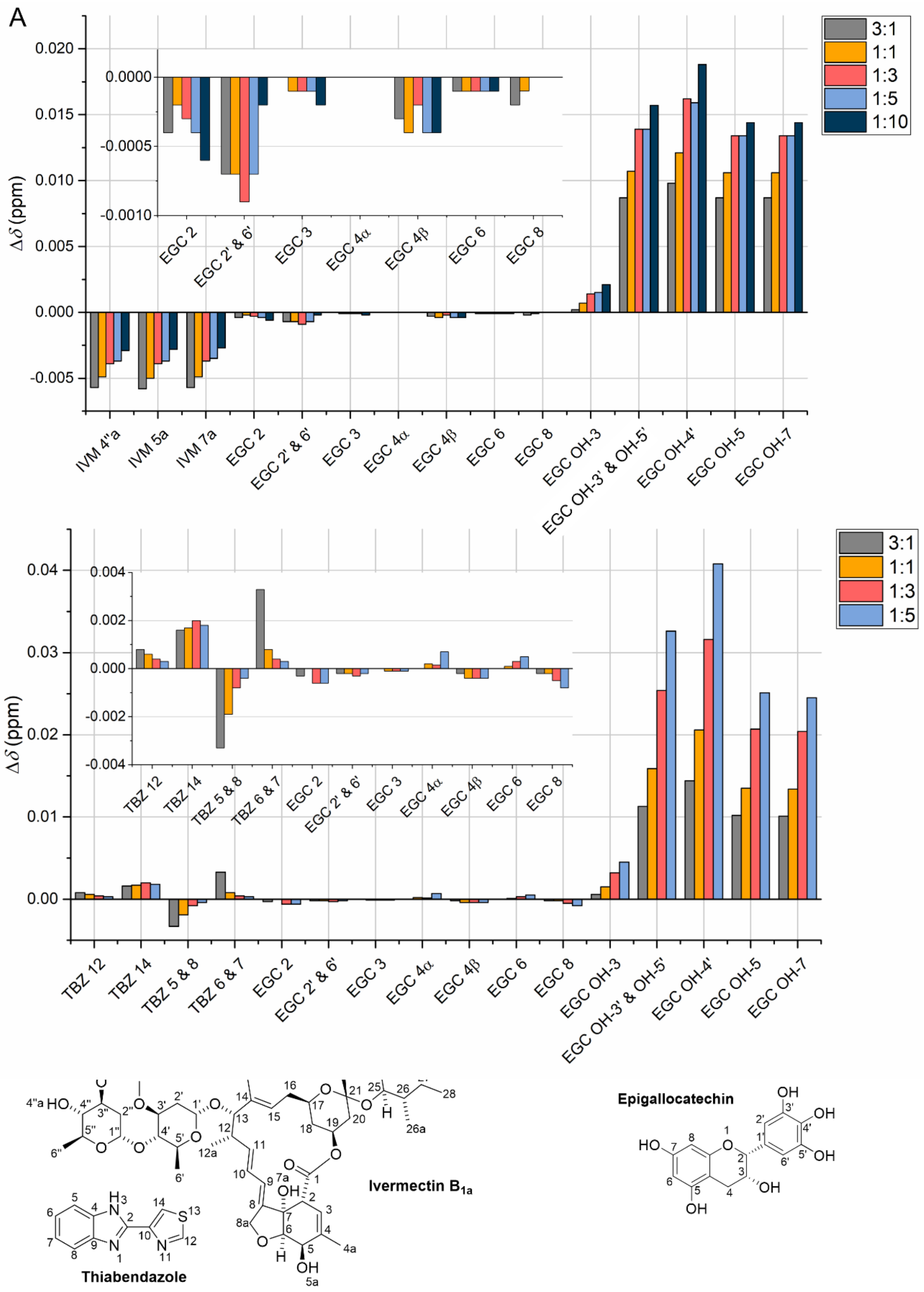

Figure S16. Changes in the chemical shifts (Δ*δ*, Δ*δ* = $\delta_{\text{mixture}} - \delta_{\text{pure compound}}$) due to the interactions between epigallocatechin (EGC) and ivermectin (IVM, A) and thiabendazole (TBZ, B) in different molar ratios of the polyphenol to the anthelmintic. Δ*δ*s are shown for selected signals of the anthelmintic and for all signals of the polyphenol. Missing data are due to overlapping signals of the components or because no chemical shift changes were observed.

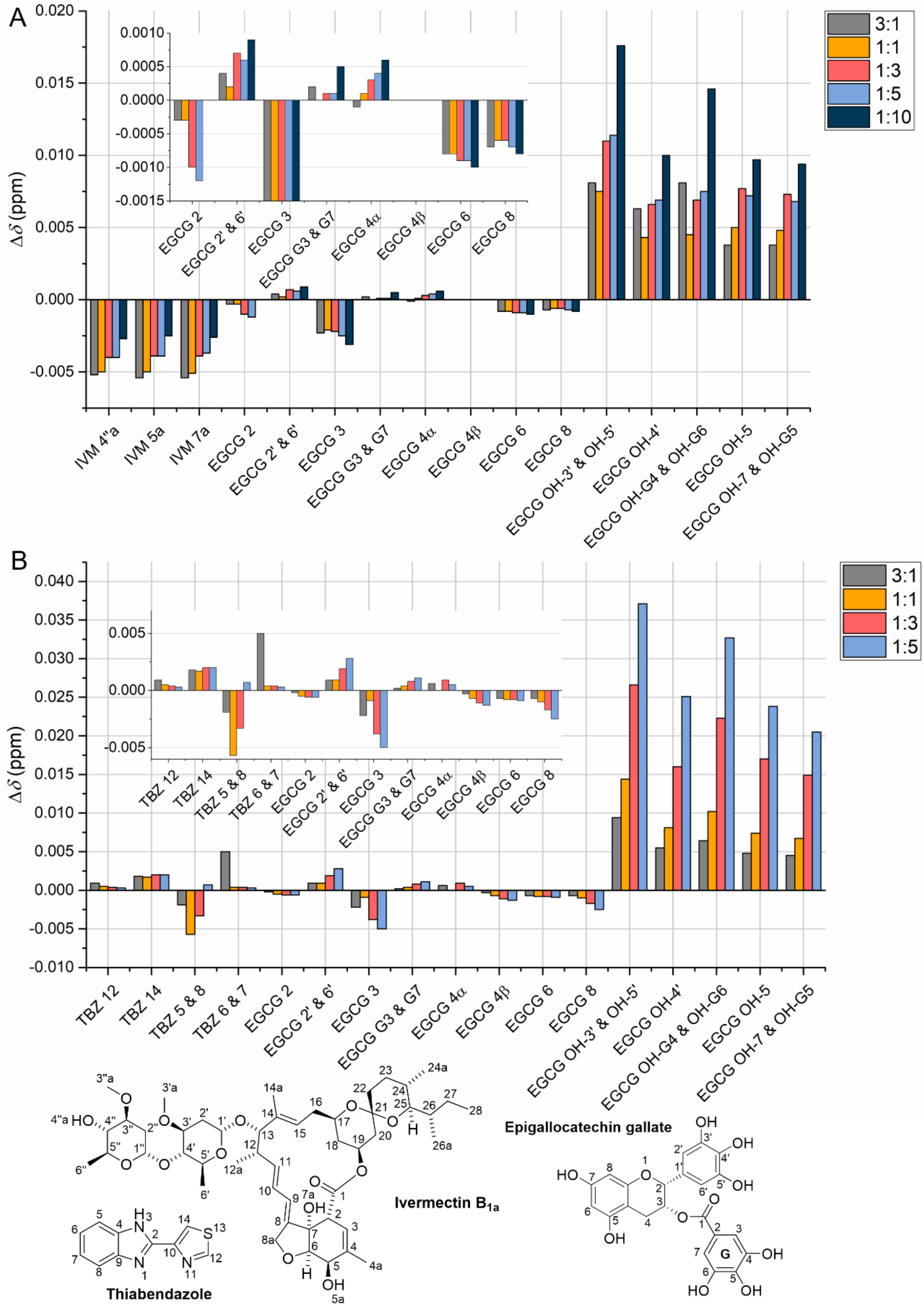

Figure S17. Changes in the chemical shifts ($\Delta\delta$, $\Delta\delta = \delta_{\text{mixture}} - \delta_{\text{pure compound}}$) due to the interactions between epigallocatechin gallate (EGCG) and ivermectin (IVM, A) and thiabendazole (TBZ, B) at different molar ratios of the polyphenol to the anthelmintic. Δδs are shown for selected signals of the anthelmintic and for all signals of the polyphenol. Missing data are due to overlapping signals of the components or because no chemical shift changes were observed.

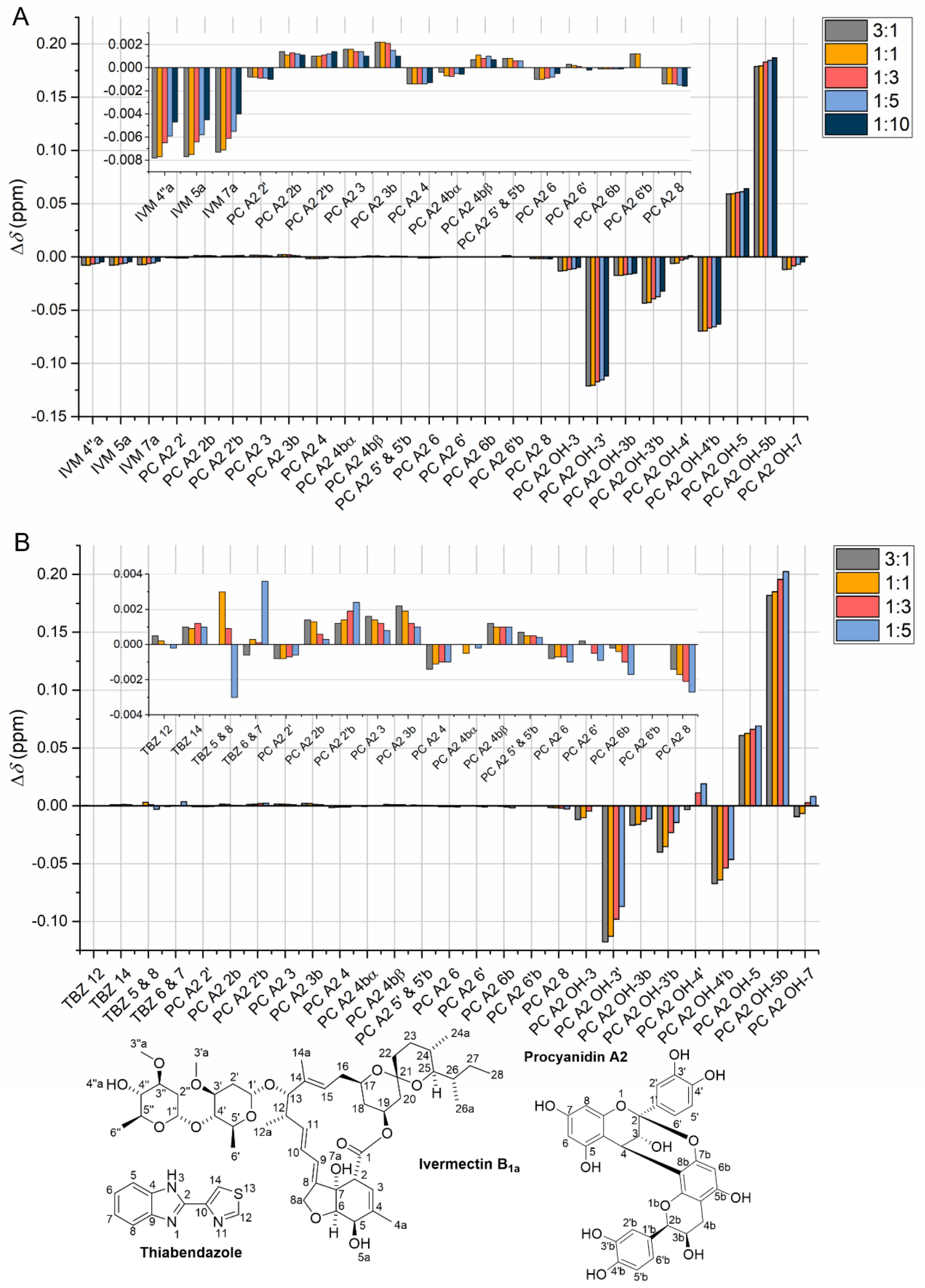

Figure S18. Changes in the chemical shifts ($\Delta\delta$, $\Delta\delta = \delta_{\text{mixture}} - \delta_{\text{pure compound}}$) due to the interactions between procyanidin A2 (PC A2) and ivermectin (IVM, A) and thiabendazole (TBZ, B) at different molar ratios of the polyphenol to the anthelmintic. $\Delta\delta$s are shown for selected signals of the anthelmintic and for all signals of the polyphenol. Missing data are due to overlapping signals of the components or because no chemical shift changes were observed.

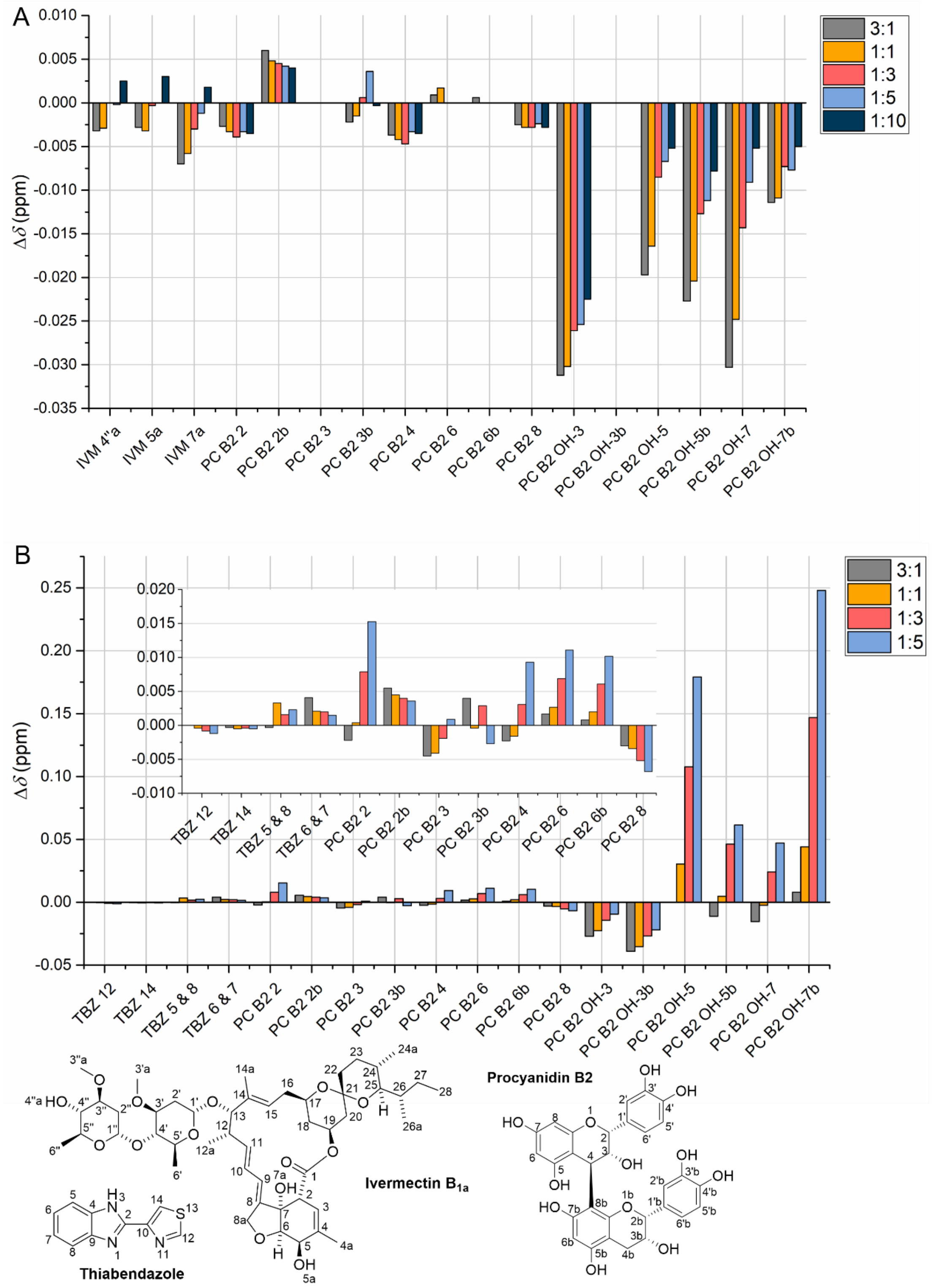

Figure S19. Changes in the chemical shifts ($\Delta\delta$, $\Delta\delta = \delta_{\text{mixture}} - \delta_{\text{pure compound}}$) due to the interactions between procyanidin B2 (PC B2) and ivermectin (IVM, A) and thiabendazole (TBZ, B) at 243 K at different molar ratios of the polyphenol to the anthelmintic. $\Delta\delta$s are shown for selected signals of the anthelmintic and for all signals of the major rotamer of PC B2, which could be characterized with certainty. Missing data are due to overlapping signals of the components or because no chemical shift changes were observed.

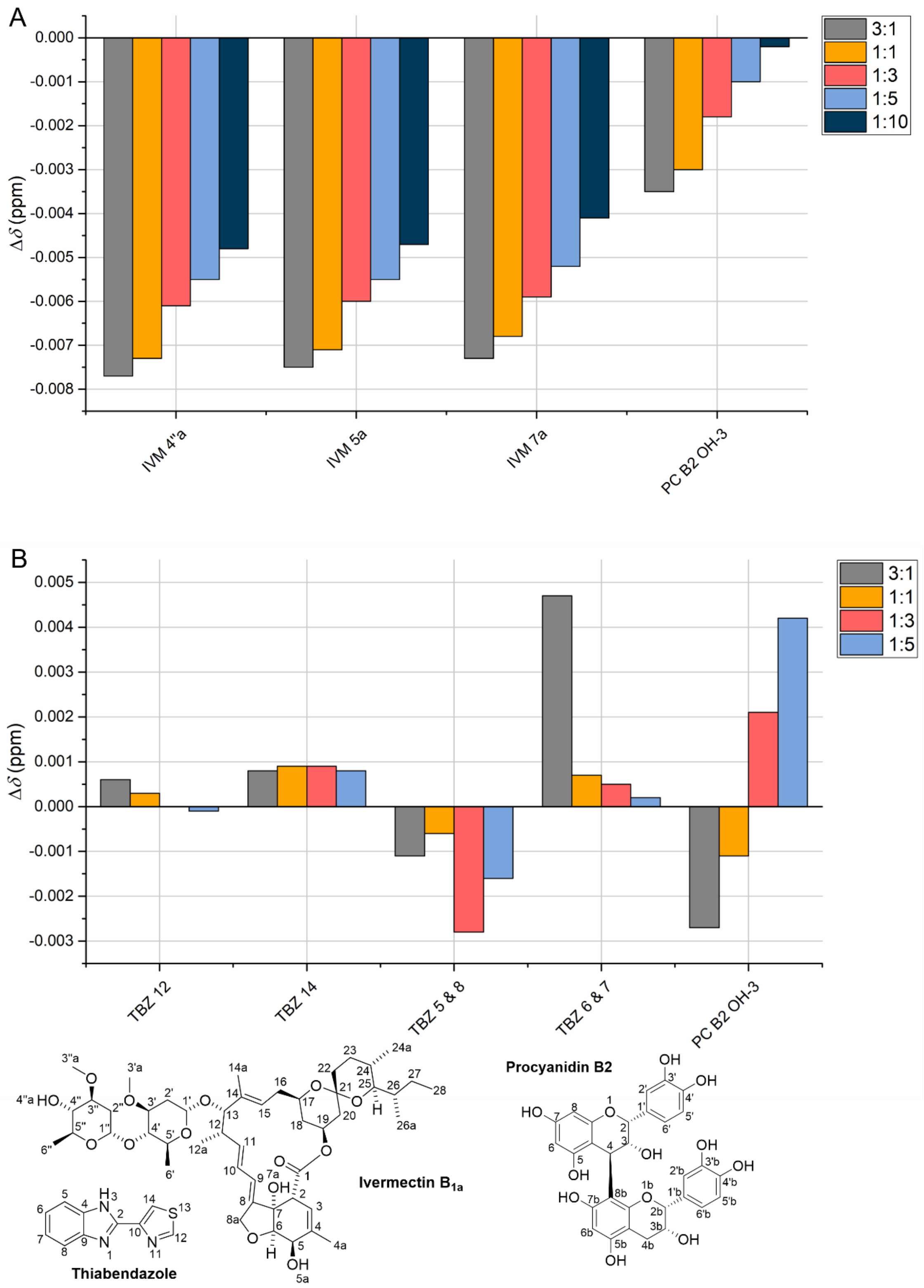

Figure S20. Changes in the chemical shifts ($\Delta\delta$, $\Delta\delta = \delta_{\text{mixture}} - \delta_{\text{pure compound}}$) due to the interactions between procyanidin B2 (PC B2) and ivermectin (IVM, A) and thiabendazole (TBZ, B) at 298 K at different molar ratios of the polyphenol to the anthelmintic. $\Delta\delta$s are shown for selected signals of the anthelmintic and for all signals of the polyphenol. Missing data are due to overlapping signals of the components or because no chemical shift changes were observed.

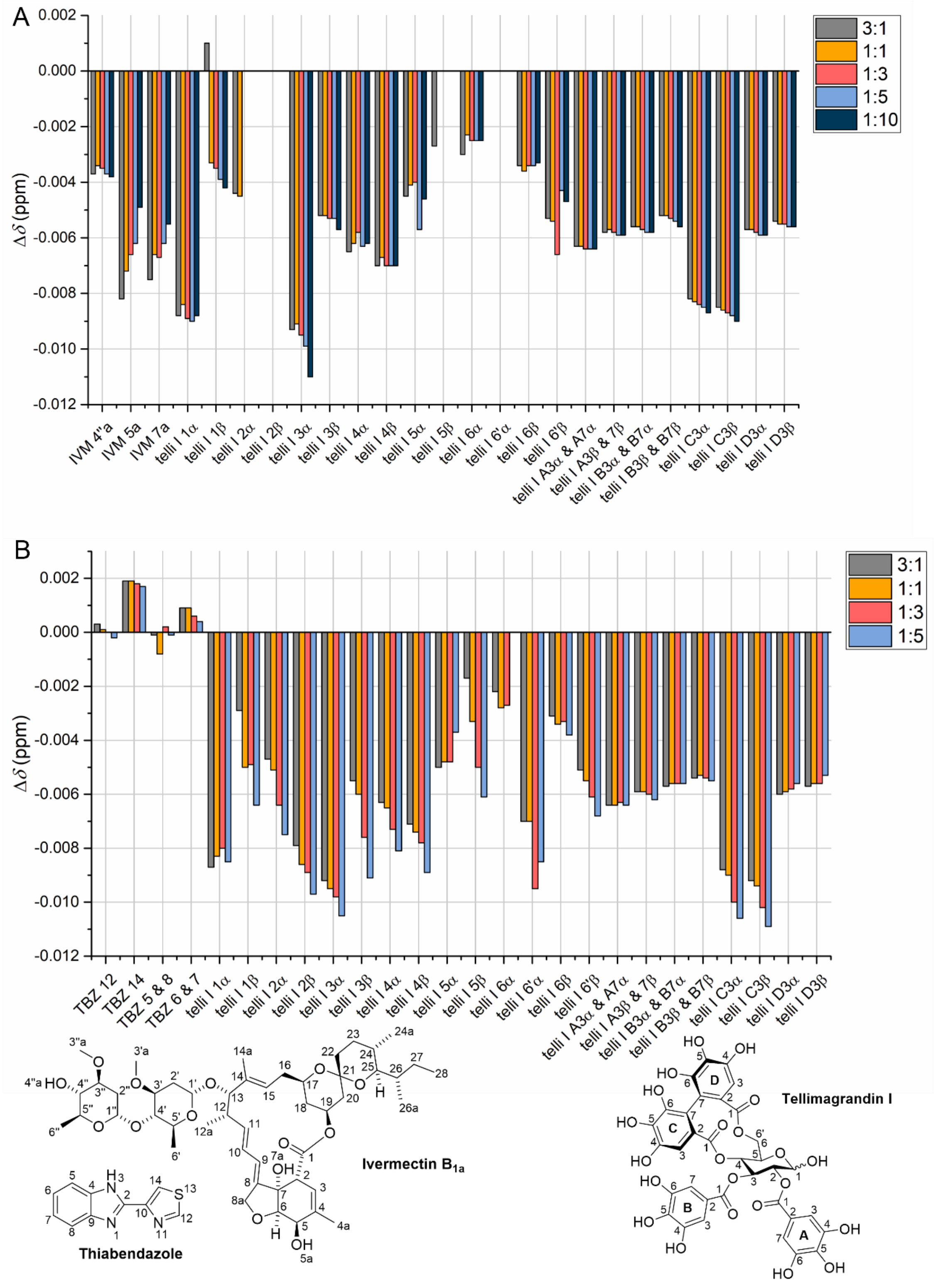

Figure S21. Changes in the chemical shifts ($\Delta\delta$, $\Delta\delta = \delta_{\text{mixture}} - \delta_{\text{pure compound}}$) due to the interactions between tellimagrandin I (telli I) and ivermectin (IVM, A) and thiabendazole (TBZ, B) at different molar ratios of the polyphenol to the anthelmintic. Δ*δ*s are shown for selected signals of the anthelmintic and for all signals of the polyphenol. Missing data are due to overlapping signals of the components or because no chemical shift changes were observed.

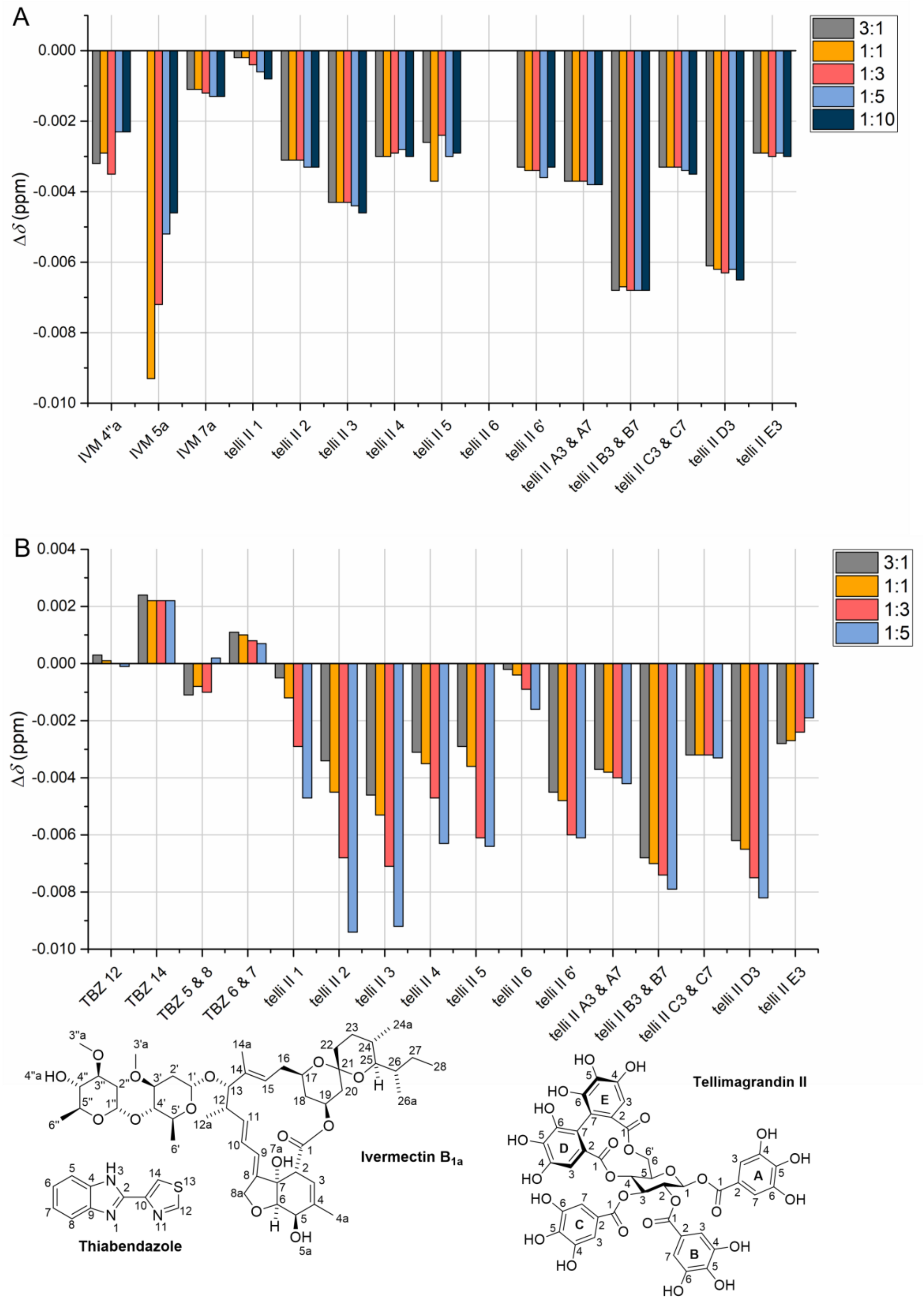

Figure S22. Changes in the chemical shifts ($\Delta\delta$, $\Delta\delta = \delta_{\text{mixture}} - \delta_{\text{pure compound}}$) due to the interactions between tellimagrandin II (telli II) and ivermectin (IVM, A) and thiabendazole (TBZ, B) at different molar ratios of the polyphenol to the anthelmintic. $\Delta\delta$s are shown for selected signals of the anthelmintic and for all signals of the polyphenol. Missing data are due to overlapping signals of the components or because no chemical shift changes were observed.

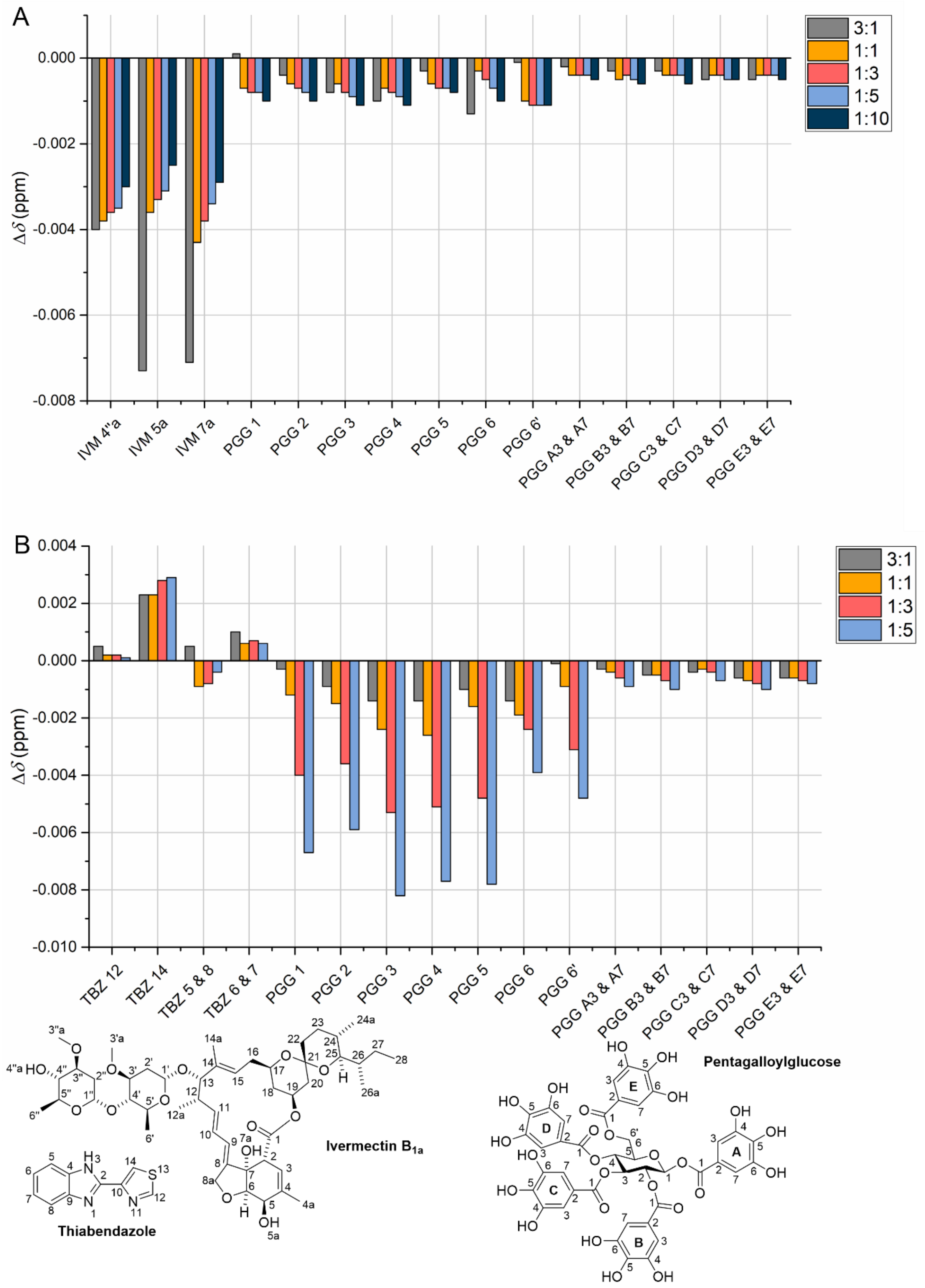

Figure S23. Changes in the chemical shifts ($\Delta\delta$, $\Delta\delta = \delta_{mixture} - \delta_{pure\ compound}$) due to the interactions between pentagalloylglucose (PGG) and ivermectin (IVM, A) and thiabendazole (TBZ, B) at different molar ratios of the polyphenol to the anthelmintic. $\Delta\delta$s are shown for selected signals of the anthelmintic and for all signals of the polyphenol. Missing data are due to overlapping signals of the components or because no chemical shift changes were observed.

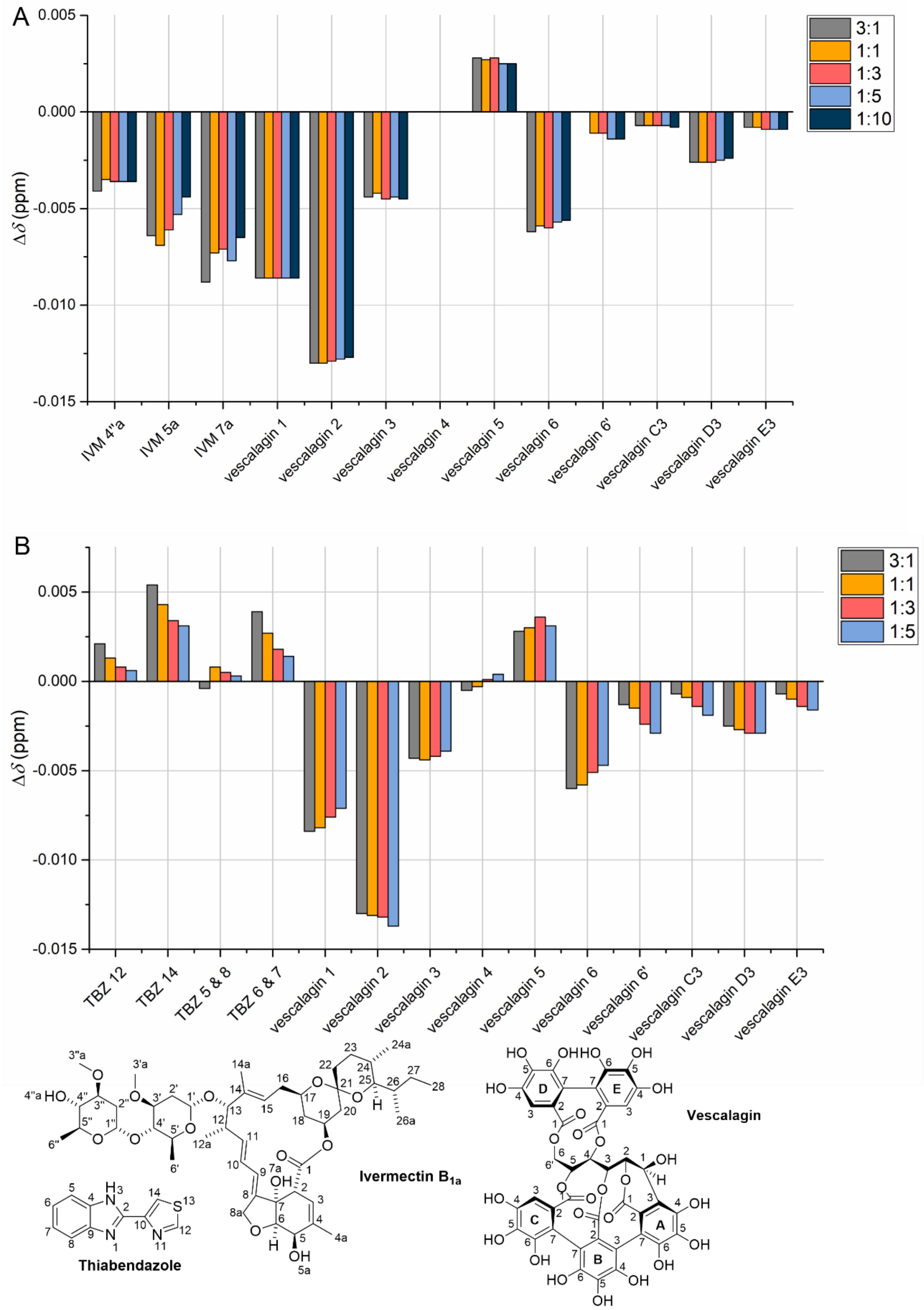

Figure S24. Changes in the chemical shifts ($\Delta\delta$, $\Delta\delta = \delta_{\text{mixture}} - \delta_{\text{pure compound}}$) due to the interactions between vescalagin and ivermectin (IVM, A) and thiabendazole (TBZ, B) at different molar ratios of the polyphenol to the anthelmintic. Δδs are shown for selected signals of the anthelmintic and for all signals of the polyphenol. Missing data are due to overlapping signals of the components or because no chemical shift changes were observed.

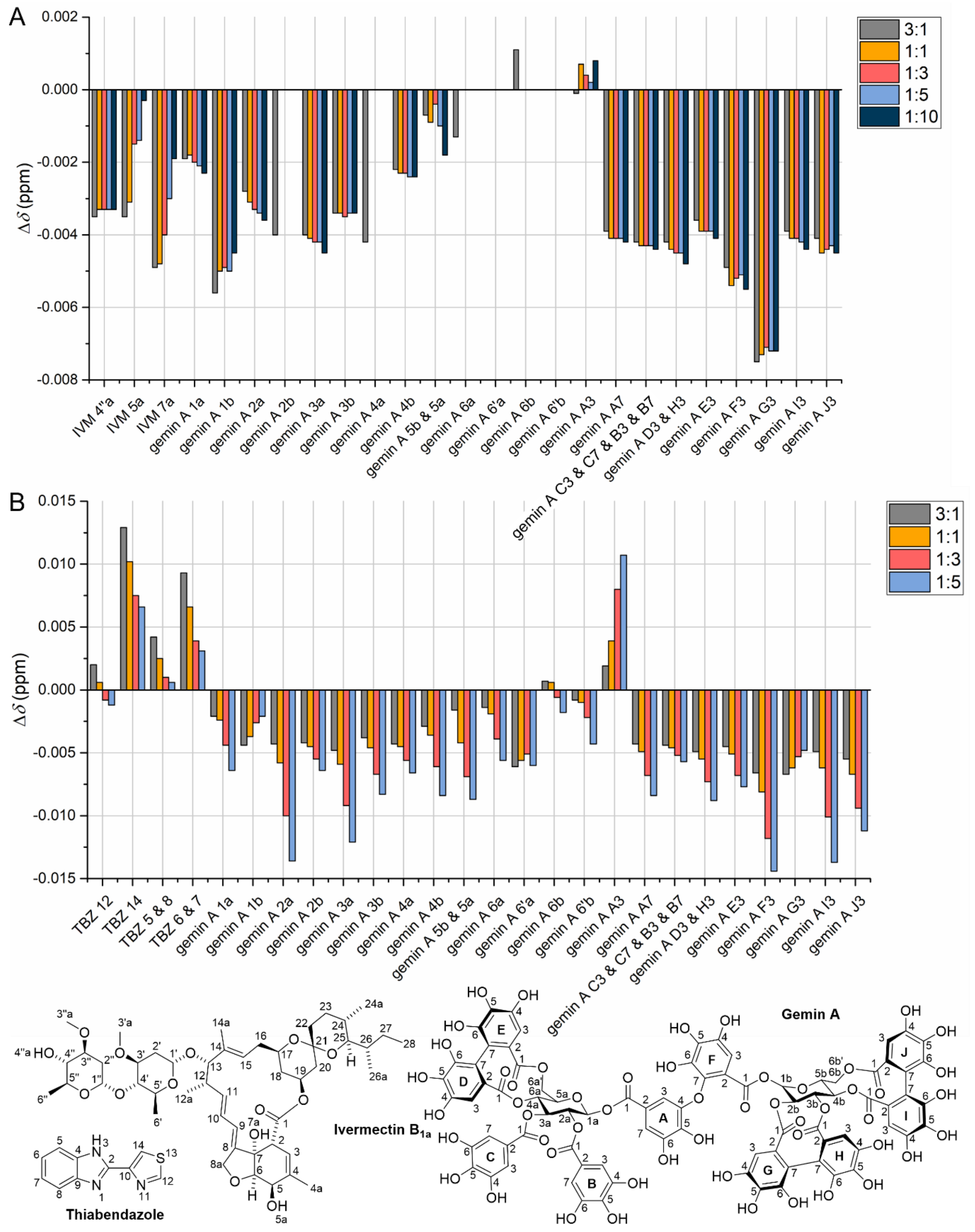

Figure S25. Changes in the chemical shifts ($\Delta\delta$, $\Delta\delta = \delta_{\text{mixture}} - \delta_{\text{pure compound}}$) due to the interactions between gemin A and ivermectin (IVM, A) and thiabendazole (TBZ, B) at different molar ratios of the polyphenol to the anthelmintic. $\Delta\delta$s are shown for selected signals of the anthelmintic and for all signals of the polyphenol. Missing data are due to overlapping signals of the components or because no chemical shift changes were observed.
